# Is it selfish to be filamentous in biofilms? Individual-based modeling links microbial growth strategies with morphology using the new and modular iDynoMiCS 2.0

**DOI:** 10.1371/journal.pcbi.1011303

**Published:** 2024-02-29

**Authors:** Bastiaan J. R. Cockx, Tim Foster, Robert J. Clegg, Kieran Alden, Sankalp Arya, Dov J. Stekel, Barth F. Smets, Jan-Ulrich Kreft

**Affiliations:** 1 Department of Environmental and Resource Engineering, Technical University of Demark, DTU Lyngby campus, Kgs. Lyngby, Denmark; 2 Centre for Computational Biology & Institute of Microbiology and Infection & School of Biosciences, University of Birmingham, Edgbaston, Birmingham, United Kingdom; 3 School of Biosciences, University of Nottingham, Sutton Bonington Campus, Loughborough, Leicestershire, United Kingdom; Abdus Salam International Centre for Theoretical Physics, ITALY

## Abstract

Microbial communities are found in all habitable environments and often occur in assemblages with self-organized spatial structures developing over time. This complexity can only be understood, predicted, and managed by combining experiments with mathematical modeling. Individual-based models are particularly suited if individual heterogeneity, local interactions, and adaptive behavior are of interest. Here we present the completely overhauled software platform, the individual-based Dynamics of Microbial Communities Simulator, iDynoMiCS 2.0, which enables researchers to specify a range of different models without having to program. Key new features and improvements are: (1) Substantially enhanced ease of use (graphical user interface, editor for model specification, unit conversions, data analysis and visualization and more). (2) Increased performance and scalability enabling simulations of up to 10 million agents in 3D biofilms. (3) Kinetics can be specified with any arithmetic function. (4) Agent properties can be assembled from orthogonal modules for pick and mix flexibility. (5) Force-based mechanical interaction framework enabling attractive forces and non-spherical agent morphologies as an alternative to the shoving algorithm. The new iDynoMiCS 2.0 has undergone intensive testing, from unit tests to a suite of increasingly complex numerical tests and the standard Benchmark 3 based on nitrifying biofilms. A second test case was based on the “biofilms promote altruism” study previously implemented in BacSim because competition outcomes are highly sensitive to the developing spatial structures due to positive feedback between cooperative individuals. We extended this case study by adding morphology to find that (i) filamentous bacteria outcompete spherical bacteria regardless of growth strategy and (ii) non-cooperating filaments outcompete cooperating filaments because filaments can escape the stronger competition between themselves. In conclusion, the new substantially improved iDynoMiCS 2.0 joins a growing number of platforms for individual-based modeling of microbial communities with specific advantages and disadvantages that we discuss, giving users a wider choice.

## Author summary

Microbes are fascinating in their own right and play a tremendously important role in ecosystems. They often form complex, self-organized communities with spatial heterogeneity that is changing over time. Such complexity is challenging to understand and manage without the help of mathematical models. Individual-based models are one type of mathematical model that is particularly suited if differences between individual microbes, local interactions and adaptive behavior are important. We have developed a completely overhauled version of iDynoMiCS, a software that allows users to develop, run and analyze a wide range of individual-based models without having to program the software themselves. There are several capability enhancements and numerous small improvements, for example the ability to model different shapes of cells combined with physically realistic mechanical interactions between neighboring cells. We showcase this by simulating the competition between filaments, long chains of cells, with single cells and find that filaments outcompete single cells as they can spread quickly to new territory with higher levels of resources. Users now have a wider choice of platforms so we provide guidance on which platform might be most suitable for a given purpose.

## Introduction

Microbes are found everywhere on Earth where conditions are suitable for life, often as microbial communities in self-organized assemblages such as biofilms [1]. They have a long evolutionary history through which they diversified into a huge number of species with fascinating characteristics and behaviors. Microbes master metabolism and thus enable biogeochemical cycles. Yet the complexity arising from the high diversity of their communities undergoing spatio-temporal dynamics makes it challenging to understand, predict and manage these communities [2]. This challenge can be best met by an integration of *in situ* observations, experiments in mesocosms and laboratory models and mathematical modeling [2].

Microbes growing in biofilms are a good example. Due to metabolic transformations of resources diffusing into the self-assembling biofilms, the aggregates become spatially structured including metabolite and resulting physiological gradients while growth leads to clonal populations. These changing environmental conditions prompt differences in gene expression, phenotype and behavior compared to planktonic cells [3]. For example, biofilm-dwelling *Pseudomonas aeruginosa* up-regulate production of extracellular polysaccharides (EPS), while *Staphylococcus aureus* biofilms up-regulate enzymes involved in glycolysis and fermentation [4]. Even in single species populations, phenotypic heterogeneity can become substantial [5,6]. Coupled to the local environmental changes, biofilm microbes experience selective pressures different to planktonic microbes. These are just a few points, but they already demonstrate the challenge of complexity in biofilm communities.

Biofilms are also a good example of insights derived from mathematical modeling, going back to the 1970s [7]. Early models treated the biofilm as a continuum in one dimension (1D), which enabled insights into substrate consumption driving the formation of gradients and diffusional fluxes and vertical stratification [8–10]. A key insight from later 2D and 3D models enabling the emergence of complex spatial structures was that the physics of mass transfer is sufficient to understand the formation of finger-like biofilm structures, arising from a positive feedback in growth where the cells at the surface of the biofilm with best access to substrate grow best so that their offspring are even closer to the substrate source and grow even better [11–13]. The detailed reconstruction of early biofilm growth observed through advanced microscopy coupled with detailed individual-based modeling of mechanical cell-cell interactions demonstrated that mechanics alone is sufficient to understand and predict early biofilm formation in *Vibrio cholerae* (before substrate gradients cause growth limitations [14]).

In such individual-based models (IbMs), microbial cells are modeled as agents, partially autonomous physical entities with individual properties and behavior [15]. This enables understanding of how these individuals affect other individuals in the community and the environment and are affected by the other individuals and the environment in turn. Properties of the community such as spatial patterns, fitness, productivity and resilience emerge from the behavior of the individuals in that community. IbMs are thus particularly suited to capture the effects of local interactions, individuality and adaptative behavior on spatio-temporal dynamics. This includes stochastic events such as dispersal and community assembly, up-regulation of genes, mutations or horizontal gene transfer. For example, Hellweger *et al*. [16] used an IbM to model the gene expression and differentiation of *Anabaena* spp. individual cells within a filament, and were able to reproduce almost all of the patterns observed *in vitro*.

To facilitate the use of individual-based modeling of microbial communities for scientists with little experience of programming, the open source modeling platform iDynoMiCS (individual-based Dynamics of Microbial Communities Simulator) was introduced [17], which we now refer to as iDynoMiCS 1. It was the result of a collaborative effort merging features of previous models into a common basis for further development. iDynoMiCS has been facilitating a range of studies and influenced the design of other modeling platforms [18–23]. Here we present, after a long phase of development and testing, a completely overhauled new version, simply called iDynoMiCS 2.0. We came closer to the original aim of making iDynoMiCS easy to use for non-programmers while substantially enhancing its capabilities and computational efficiency. Key new features and improvements are: (1) Enhanced ease of use right from the start, from using a simple guided java installer, lack of dependence on other software installations, a graphical user interface (GUI) for running simulations, editing model specification (protocol) files, unit conversions, data analysis and visualization of live results or re-loaded past results, a collection of model examples and online wiki for guidance, autonomous adjustments for solver convergence. (2) Increased performance and scalability enabling simulations of up to 10 million agents in 3D biofilms. (3) Kinetics of chemical or agent-catalyzed reactions can now be specified with any user-chosen arithmetic function. Local or intracellular conditions can be incorporated in these expressions enabling adaptive behavior such as metabolic switching or change in kinetics due to mutations. (4) Agent properties can now be assembled from orthogonal modules giving the user pick and mix flexibility. The same is true for processes. Thus, the complexity of agents or processes can be adjusted to fit the modeling purpose. Due to the modular structure, it has become easier to implement novel functionality. (5) Force-based mechanical interaction framework enabling attractive forces and non-spherical agent morphologies, which was impossible with the shoving algorithm. We showcase this new feature in a case study demonstrating how the fitness of filaments benefits from escaping competition.

## Model development and description

The description of iDynoMiCS 2.0 and the case studies presented in this paper follow the ODD (Overview, Design concepts, Details) protocol for describing individual- and agent-based models [24,25]. However, iDynoMiCS 2.0 is not one specific model but a platform to facilitate the specification of a broad variety of models. Hence, this section aims to provide a general description of the platform but cannot cover all possible models that could be simulated. Thus, we also provide detailed model-specific ODD descriptions together with the presented case studies.

### Overview

The purpose of iDynoMiCS 2.0 is to facilitate the simulation of large groups of individual microbes and their interactions in a microbial population or community, either in a well-mixed chemostat-like or a spatially structured biofilm-like compartment. The aim is to study and predict how the interactions and properties of individual microbes lead to emergent properties and behaviors of microbial communities.

### Entities, state variables and scales

Entities, state variables and scales may vary from one model implementation to another. In a typical implementation, microbial cells are the principal agents. They can mediate both chemical and physical activities. Agents can have any number of properties and behaviors. Typical properties are position, mass, density, shape, composition and metabolic reactions. Typical behaviors are cell growth, division, death, extracellular polymeric substance (EPS) production and excretion. iDynoMiCS 2.0 refers to properties and behaviors of an agent as the “aspects” of an agent. Shared aspects can be set-up as a module and reused for all agents sharing these aspects. A typical agent is one or multiple orders of magnitudes smaller than the computational domain.

The simulated space (computational domain) in which agents reside is called a compartment. There are two types: spatially explicit compartments in 2D or 3D to simulate microbial assemblages such as biofilms and well-mixed compartments used to simulate the dynamics of a bulk liquid without agents or a planktonic community with or without inflows and outflows (batch, fed-batch or chemostat); these compartments are assumed to have no spatial structure and thus the concentrations of chemical species and agents are homogeneous.

Well-mixed and spatially explicit compartments may be connected to simulate how bulk and biofilm dynamics are coupled. Compartments can have multiple properties including: boundaries, physical dimensions, volume and a scaling factor. This scaling factor is used to translate between the size of a simulated representative volume element and the actual size of the system, it allows a smaller simulated compartment to represent a larger entity. There are no hard restrictions on compartment size, however, compartments typically have lengths up to a millimeter. Agents can only reside in a single compartment at any one time, but they can be transferred or move between spatially explicit and well-mixed compartments.

Dissolved chemical species in iDynoMiCS 2.0 are referred to as solutes. Compartments can contain any number of solutes. In well-mixed compartments, a solute is represented as a single concentration. In spatially explicit compartments, solutes are represented as 2D or 3D concentration fields in a Cartesian grid. The distance between grid nodes is referred to as the resolution, typically one to a few micrometers.

### Framework structure

**Fig 1:**
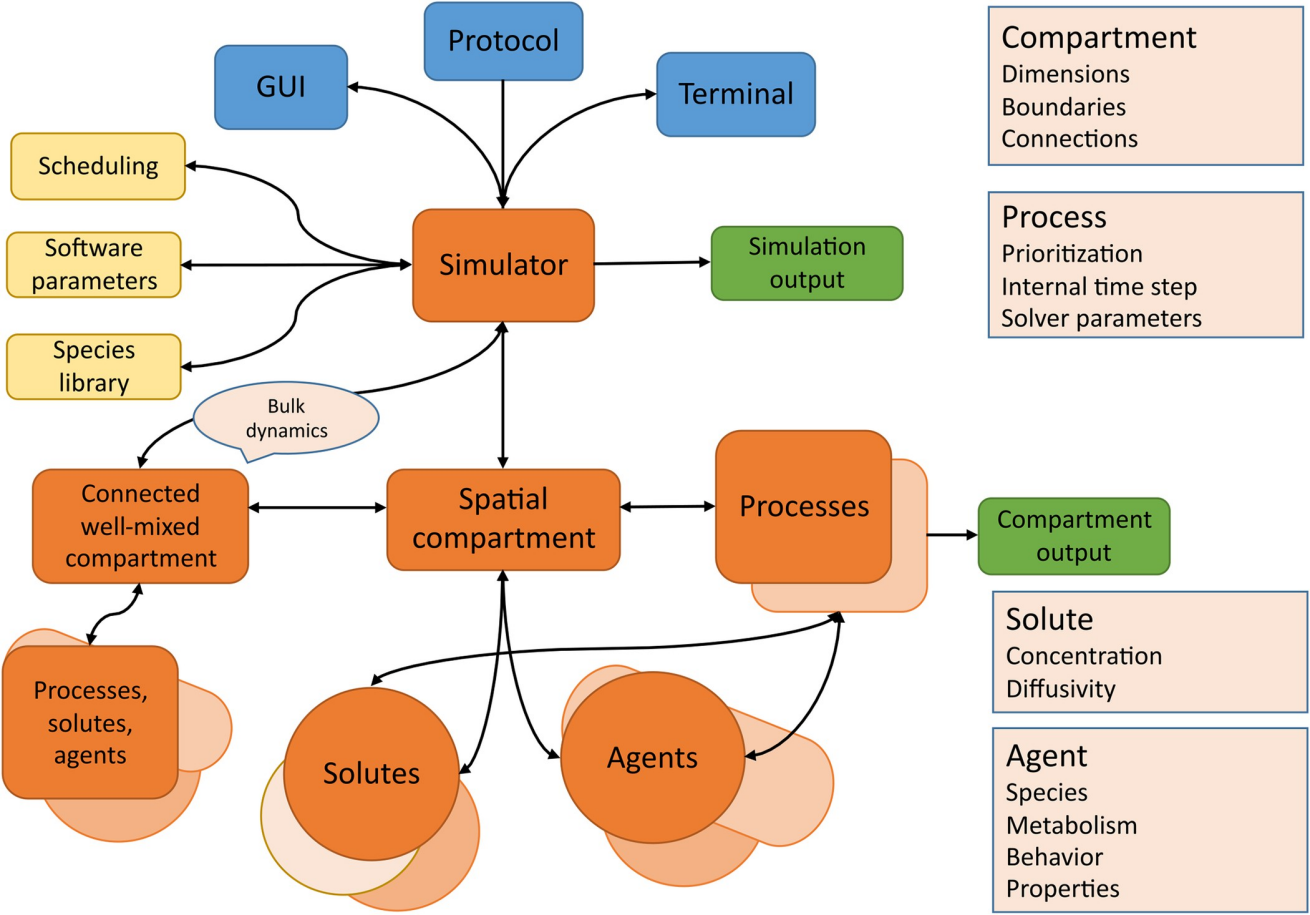
The basic structure of an iDynoMiCS 2.0 model. Interaction with the program takes place through the GUI or command line terminal. A protocol file specifying a model can be loaded to initialize the simulator. If parameters are missing from the protocol file, a default is loaded or the user is queried if no default exists. Scheduling ensures predictable handling of the compartments and the order of processes occurring within them. A species library is kept such that properties and/or behavior that are identical for agents of the same species can be looked up from the library. The simulator further ensures that the model state is saved at the end of each global time step. Spatially explicit and well-mixed compartments can be connected. Solute concentration fields are stored as matrices, which include local solute concentrations, local diffusivity and reaction rates. The collective of agents represents the biofilm, agents may have many properties depending on user specifications, basic properties are species, mass and position of the agent. Processes act upon the information in the model system and describe the processes occurring in the model such as mechanical interactions or diffusion, or generate output from the active model state. For this figure, colors are used to distinguish between the different elements of iDynoMiCS 2.0. The core elements are orange, input elements are blue, output elements are green and helper algorithms and data structures are yellow.

In iDynoMiCS 2.0, models are structured, specified and instantiated hierarchically. The basic structure of a typical scenario is presented in Fig 1. The “Simulator” is the root of any model implementation. It loads the model specification from a protocol file provided by the user either through the GUI or the command line terminal. General software control parameters are managed at the simulator level, as well as the scheduling of sub-models, the management of compartments and the storage of species modules (reusable sets of aspects). The simulator steps through its compartments and saves the model state at each global time-step. A compartment stores its shape and size, solutes and agents in the compartment and processes occurring within the compartment. Agents and processes store their properties as aspects. This layered structure of model input provides a level of modularity to the iDynoMiCS 2.0 software and model implementations.

Modularity enables flexible combination and facilitates software maintenance and development. An iDynoMiCS 2.0 model is formulated as distinct modules describing specific parts of the model such as a compartment, process or an agent. An example model description is included in the section *input*. These modules can be referenced to add the same object, property or process in another compartment or agent. For example, multiple agents of different species can implement the same module describing cell shape but implement a different module describing metabolism.

Within the software, modularity is implemented through the use of software interfaces and abstractions. These software interfaces ensure common functionality such that loading or storing of data, scheduling and initiation are handled in the same way for any software class implementing these interfaces. This concept makes it easier to add new features and extensions to iDynoMiCS 2.0, since an extension can be integrated into the framework without additional work to handle initiation, data handling, scheduling and other auxiliary functionality (Box A in S1 Text).

Support for arithmetic and basic logic expressions provides flexibility to iDynoMiCS 2.0 models. Users can simulate any type of kinetic or physical interaction model that can be described by a standard arithmetic expression. Logic expressions are particularly useful in models with biological switches or thresholds. Typical examples are metabolic or morphological switches. Since any kind and number of aspects can be changed, the characteristics of an agent can completely change at runtime. Logic expressions can also be used to filter agents matching specific criteria, which can be useful for further analysis, or to color agents based on their properties. Logic expressions may incorporate arithmetic expressions.

### Process overview and scheduling

Many processes can take place in a microbial community, often simultaneously. To capture these processes in a computer model, they are represented by simplified mathematical models, discretized and handled in an asynchronous fashion. An iDynoMiCS 2.0 simulation steps through a number of global time-steps. Within each global time-step and for each compartment, sub-models describing activities occurring in the microbial community are executed. Biological, chemical and physical processes included in a model depend on the purpose and design of the model. However, most simulations include: physical interactions, (bio-)chemical reactions, diffusion of chemical species, microbial growth and cell division. A detailed overview of implemented submodels follows later. The user may also include helper processes that do not alter the model state, but instead perform other useful functions at user defined time intervals such as model analysis, reporting and generation of graphical output.

During one global time step, a list of processes, defined and scheduled by the user, are executed. After this, the model state is saved and any updates involving connected compartments occur, this may include updating the concentration at a boundary and the transfer of agents from one compartment to the other. Each process is assigned a time step size and a priority by the user, if not, the global time step and order of occurrence in the protocol file are adopted as time step and priority. A process can thus operate at a time interval independent of the global time step although it is a good idea to align the time steps to the global time step. A process may further divide its specified time step into smaller internal time steps if the involved solvers or algorithms require this. Processes are handled in a specific order; the process time step is used to determine which process is due first. If multiple processes are due simultaneously, the process priority is used to determine the order. Box 1 shows a scheduling example as implemented in the biofilms promote altruism case study presented later.

**Box 1. Process schedule for the biofilms promote altruism case study within a single global time step**

1. (Bio-)chemical reactions and diffusion
  - Update solute grids and agent masses (e.g. (bio-)chemical conversion)
  - Update solute boundaries
2. Agent updates
  - Cell division
  - Differentiate (switch between sphere or rod shaped if applicable)
  - Update cell size
3. Mechanical relaxation
  - Update cell positions and mechanical stresses until relaxation criteria are met
4. Compartment reporting
  - Density grid (csv)
  - Compartment summary (csv)
  - Graphical output (pov/svg)
5. Save simulation state (xml/exi)

### Design concepts

#### Basic principles

Microbes are modeled as individual particles that interact with their environment. Mass is conserved and thus the mass balance of any ‘element’ in the model system should be closed.


in+production−out−consumption=0 (1)

#### Emergence

System-level phenomena such as the distribution of microbial agents, the spatial structure of a biofilm, and chemical conditions all emerge from interactions between agents and their local environment. This local environment in terms of interactions with other agents is any agent (or surface) within a specified interaction distance (e.g., larger for plasmid transfer than collision) and in terms of the environment are the solute concentrations in the grid element the agent is located in.

#### Adaptation

iDynoMiCS 2.0 models can include adaptation. By sensing the local environment or internal states, an agent may change its characteristic using the differentiate aspect. Adaptation can also be stochastic, where heritable stochastic changes in an agent’s characteristics can lead to selection of the lineage best suited to survive or thrive in the simulated environment.

#### Objectives

Agents can have objectives. For example, agents may have the objective to move to a region with higher substrate availability (chemotaxis).

#### Learning and prediction

Although microbes have no brain, they can have memory and process signals. For example, chemotaxis requires memory. Also, event occurrences may be stored as an aspect of the agent and influence the behavior or characteristics of an agent.

#### Sensing

Locally sensed nutrient concentrations can affect growth rate or adaptation. Neighboring cells may affect the spatial direction of cell division in filamentous agents or affect physical displacement. A combination of the above supports microbial behaviors such as dormancy or chemotaxis.

#### Interaction

Agents interact with their local environment by consuming and producing solutes, including extracellular enzymes, or particles such as EPS. As a consequence, they indirectly interact with neighboring agents, leading to competition, cooperation or communication. The agents further interact with their physical environment by pushing or pulling other physical entities, including other agents as well as physical surfaces in the computational domain.

#### Stochasticity

Model implementations can include stochastic processes, examples are allocation of biomass to daughter cells upon cell division, placement of daughter cells relative to the position of the mother cell, stochastic movement (for example Levi flight), and mutations or other random perturbations. A random seed is saved so simulations can be continued with consecutive random numbers or repeated with an identical series of random numbers if desired.

#### Collectives

Microbes may aggregate actively through motility coupled with communication and the expression of surface proteins or passively through cell division and the production of an extracellular matrix [26]. Consequently, agents can form a local collective of unrelated or related cells. Moreover, communication can coordinate collective action that does not require aggregation. iDynoMiCS 2.0 has the basic building blocks to model coordinated collective behavior such as quorum sensing.

### Observation

The model state is saved at the end of each global time-step. Additional compartment output can be saved, including physical and biological states of all agents and the chemical state, at any given time and location in the simulation.

### Initialization

The initial state of a simulation is highly flexible and should be provided by the user. This includes solute concentrations as well as positions and states of agents, which may be based on experimental data or generated to investigate hypothetical scenarios. iDynoMiCS 2.0 includes several helper classes to generate initial states. A ‘random spawner’ can be used to randomly distribute large numbers of agents of a certain type over a pre-defined region, applied in the initialization of the stress test and Benchmark 3 case study. A ‘distributed spawner’ has a similar function but distributes agents regularly at a pre-defined interval, applied in the initialization of the biofilms promote altruism case study. It is also possible to manually define an initial state for any individual or to utilize a previous simulation outcome as an initial state for a new simulation, for example to implement a perturbation.

### Input

All simulations are initiated using iDynoMiCS 2.0 protocol files. They follow the logical structure of the model setup and are structured using the Extensible Markup Language (XML). Both XML and EXI (Efficient XML Interchange) files can be used. It is recommended to include units when specifying parameters in the protocol file, units are converted to iDynoMiCS’s base unit system, which avoids unit conversion mistakes. An abbreviated example protocol is included in Box 2.

**Box 2. An abbreviated protocol file showing the hierarchical structure**

Setting up a basic protocol is relatively simple and supported by the GUI. In this example, 30 copies of an agent of species “bacterium” are added to a rectangular domain. Bacterium is defined by the reusable “coccoid” aspect highlighted in green as well as a growth reaction which is only used for this species. The coccoid module describes basic physical properties and behavior of a generalized coccoid including agent density, division mass, etc. Bacterium contains the “reactions” aspect which is a list of all reactions the agent can catalyze (in this case only the crucial growth reaction). The reaction node includes an arithmetic expression defining the growth kinetics based on the local solute concentrations of two solutes (carbon and oxygen) and associated kinetic constants and stoichiometry. The protocol further describes the properties of the compartment, the solutes and processes to be loaded. The full protocol file is just 60 lines and included in S1.7.

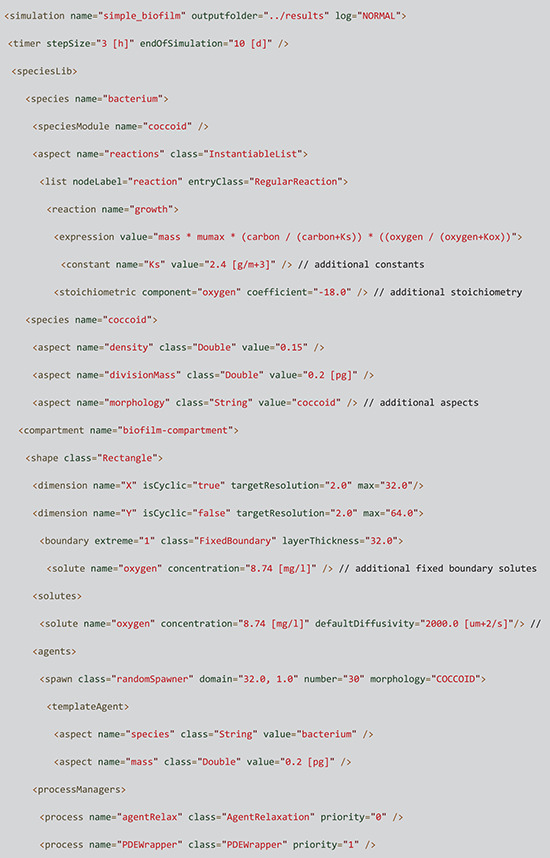

### Output

The model state is saved as XML or EXI file to reduce file size after each global time step and follows the same structure as a protocol file. The output also includes all information required to restart the simulation. It is also possible to save additional output per compartment to facilitate later analysis or visualization. Visual output includes SVG or POV formats to render the agents in a compartment. The hue, saturation or brightness of the agent can be adjusted based on its properties to convey additional information about the agent’s state. A CSV file listing agents with key properties of interest can also be produced, this list can be filtered to only include agents matching specific criteria. Further, the density of agents in the compartment can be reported for all agents or those matching specific filters (such as belonging to a certain species). Finally, a summary with key statistics on the compartment can be written such as mean solute concentrations, total agent counts and masses, agents matching specific criteria, etc. The summary is useful to quickly plot time series data.

### User interface

Simulations can be loaded and started through the command line or a GUI (Fig H in S1 Text), which may be used to review, edit or create protocol files before running them. During the simulation, the GUI provides key information on the simulation state (such as substrate concentrations, species abundance, convergence of the reaction diffusion solver, etc.). The spatial domain can be rendered directly to display agent distributions and concentration gradients. Lastly, the GUI can be used to extract key data from iDynoMiCS 2.0 output files, convert between EXI and XML files and convert numbers between different unit systems including SI and iDynoMiCS 2.0 base units.

### Submodels

Here we describe key submodels currently implemented in the iDynoMiCS 2.0 platform. Specific model implementations may utilize a subset of these submodels or alternative submodels that extend capabilities further.

#### Physical representation of agents

**Fig 2:**
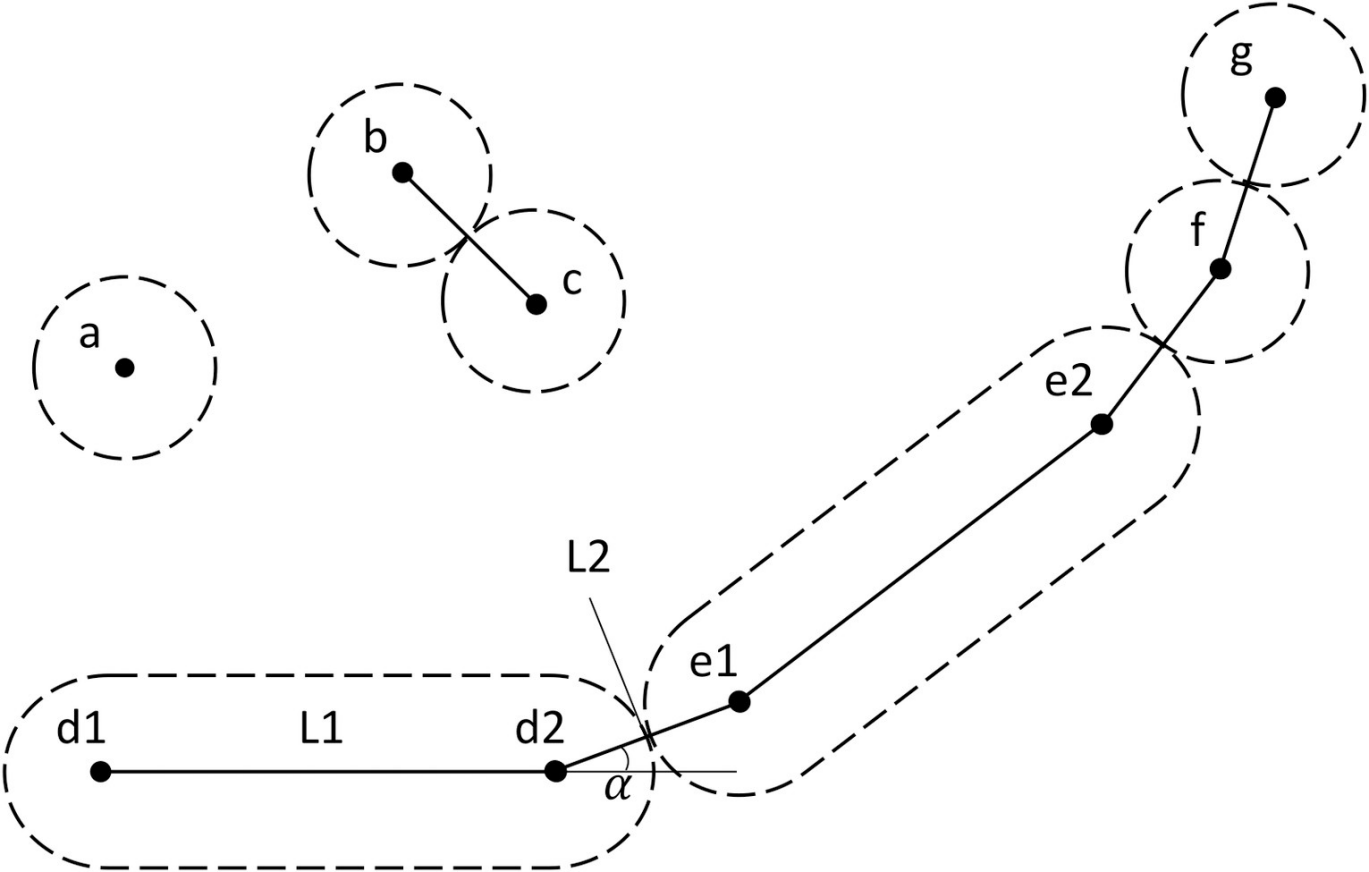
Different agent shapes in iDynoMiCS 2.0. Dashed lines indicate sphere-swept volumes of ‘dots’ or line-segments. Dots are mass-points indicating position and orientation of agents. Solid lines indicate mechanical interactions between points (forces between points modeled as springs): Collision interaction (b-c), spine interaction responsible for the rigidity of rod-shaped agents (d1-2 and e1-2), connecting interactions (d2-e1, e2-f, f-g). α is the angle between two elements of a filament. This angle can be counteracted by a torsion spring applying forces on d1, d2 and e1. L1 and L2 are the moment arms. The torsion spring applies force until the angle α reaches 180°, aligning the three points.

Microbial cells in the model have position and extent in 3D continuous space, constructed from points and swept-sphere volumes (Fig 2). Specifically, spherical cells dubbed “cocci” are constructed from a single point with a spherical volume, rod-shaped cells dubbed “bacilli” are constructed from two points connected by a line-segment and a swept-sphere volume along the line-segment, filaments are represented as a chain of either one or both. Every point has a mass associated with it. As cells grow, they may divide when a threshold mass is reached. Upon division, mass and other compounds are divided between the original cell and the newly created cell. A user supplied value is used as a coefficient of variation to obtain a random value from a truncated (+/- 2σ) normal distribution with the mean equal to 50% of the mass of the original cell. The placement of the newly formed cells is dependent on their morphology, this process is described in S1.14.

In order to simulate 2D models, a number of assumptions and adjustments have to be made. An implicit third dimension (z) is required to retain consistency for physical units such as volume or concentration, in iDynoMiCS 2.0 this third dimension is 1 μm thin. The 2D agent shapes are extruded into this virtual dimension, thus their pseudo 3D shapes have a uniform cross-sectional area and a thickness of 1 μm.

One side effect of simulating biofilms in 2D results from the constraint that the length of the virtual third dimension has to be identical for all agents. As a 2D agent can only grow in two dimensions, the relationship between the biomass and the radius and/or length of a 2D agent differs from that of a 3D agent. This can result in unwanted agent size effects, especially for agents with radii much smaller or larger than the fixed 1 μm length of the third or z dimension. iDynoMiCS 2.0 can scale the density of agents in order to match agent diameters and lengths in 2D simulations to those that an agent of equal mass would have in a 3D simulation. This method is described in detail in S1.12. This method is not a perfect solution as it will introduce new side effects, the local shifts in biomass concentrations will increase nutrient competition for large agents whilst decreasing it for small agents. If there are large differences in agent size, it is recommended to test validity of 2D simulations with 3D simulations.

Another side effect of the translation from 3D to 2D is the higher density of circle packing compared to sphere packing [27]. To mitigate this effect, Clegg *et al*. [27] proposed a density scaling factor equal to the ratio of the maximum packing density of spheres over the maximum packing density of circles $\frac{{ŋ}_{sphere}}{{ŋ}_{circle}}\approx 0.82$ for 2D simulations. This can be applied in iDynoMiCS 2.0 simulations by multiplying the densities that would be used in a 3D simulation by this scaling factor. Appropriate scaling factors for other agent shapes or mixtures of agent shapes are unknown.

Additionally, biofilm structure and development can be affected in 2D simulations. Vertically stratified biofilms with chemical gradients from substratum surface to the biofilm-liquid interface dominating, or biofilms with gradients in the second dimension, parallel to the substratum surface, that are equivalent in the third dimension which is also parallel, can be accurately modeled in 2D. However, biofilms that form finger-like or other superstructures become artificially substrate limited due to the lack of mass transport in the third dimension. Consequently, these superstructures will form under different environmental conditions in 2D versus 3D.

### Mechanical interaction framework

In the original iDynoMiCS 1, agent overlap was resolved with a shoving algorithm. In iDynoMiCS 2.0, mechanical interactions between agents (or with surfaces in their environment) as well as between different points within the same agent are based on forces. This Force-based Mechanics (FbM) framework builds on the mass-spring models of Janulevicius *et al*. [28], Celler *et al*. [29] and Storck *et al*. [30] but is no longer limited to spring forces. One shoving algorithm variant is still available as an alternative or for comparison with iDynoMiCS 1.

Before an interaction force can be calculated, the interaction needs to be detected. Direct links between two points of the same agent are stored as an aspect of the agent. Additionally, neighboring entities are found efficiently through a search of the quad- or octree that keeps agents spatially sorted. Through collision detection, as described and implemented by [30,31], physical interaction between neighboring agents is tested. The distance between two objects (which can be negative) is used in a force model to calculate the resulting force of the interaction. The quad-, octree and collision detection algorithms were modified to work with periodic boundaries.

#### Forces

The FbM solver exploits the fact that under conditions of very low Reynolds numbers, inertial forces on a particle become negligible [32,33]. Because of this, the sum of all forces applied to the mass-point (the net force ∑*F*_*p*_) can be assumed to balance the mass-point’s drag force *F*_*D*_ (*F*_*D*_ = ∑*F*_*p*_) nearly instantly. By applying Stokes’ law, the terminal velocity of the mass-point *v*_*t*_ can be obtained, under low Reynolds number conditions the mass-point reaches this velocity nearly instantly and thus we can assume *v*≈*v*_*t*_ when Re << 1 (Eq 2) [32,33]. This simplification effectively halves the number of ordinary differential equations that need to be solved.


$$v\approx v_{t}=\frac{\sum F_{p}}{6\pi r_{c}\mu_{f}}$$ (2)

With *r*_*c*_ the radius of the cell and *μ*_*f*_ is the dynamic viscosity of the fluid.

The mass-point’s velocity, and by extension the point displacement, is resolved using a forward Euler method or the second-order Heun’s method [34].

Multiple types of interactions may lead to a net force being exerted on any given point. These forces may be due to collision, close proximity repulsion or attraction, attachment and internal structure. A force model for these interactions can be provided through the protocol file. Default models are used if no model is provided. The default force model for agent collision is the Hertz soft sphere model [35] (Eq 3), where r_eff_ is the effective radius, E_eff_ the effective Young’s modulus and ξ the agent overlap.


$$F=\frac{4}{3}\sqrt{r_{eff}}E_{eff}\xi^{3/2}$$ (3)

Rod-shaped cells and filaments have permanent connections between points (L1 & L2 in Fig 2). By default, these connections are modeled as linear springs following Hooke’s law (Eq 4), where k is the spring constant and *δl* the difference between the current length and the rest length.


F=kδl (4)

An internal force can also be specified to resist bending, for example for segments of a filament (Angle α in Fig 2). The default model for such torsion springs is the angular form of Hooke’s law (Eq 5), where κ is the spring constant, *δ*ϴ the difference between the actual angle and rest angle, and L the length of the momentum arm.


$$F=\frac{\kappa \delta ϴ}{L}$$ (5)

Forces for agents in proximity can also be specified, for example, close range repulsion and/or attraction, but are not applied by default.

#### Reactions

A reaction transforms chemicals; these chemical species may be modeled explicitly as solutes or types of the structured biomass within cells (e.g., regular biomass and storage compounds), or implicitly and so left out of the model description (e.g., water in aqueous environments). Reactions have a rate and stoichiometry. They may be catalyzed by agents or occur independently of agents in the environment.

Reaction rates may depend on the concentration of certain reactants, such as solutes or biomass, and other variables, such as temperature. Within iDynoMiCS 2.0, rate equations can be expressed through any type of arithmetic expression, which allows the use of almost any kinetic model rather than a predefined set of kinetic expressions including the commonly used Monod type kinetics (Eq S3). Many variations of Monod kinetics and alternative kinetic models exist [36,37]. While the kinetic parameters are typically obtained experimentally, models that utilize thermodynamic relationships have been developed [38–40]. These thermodynamics-based models are typically formulated following a Monod type expression, or through the Herbert-Pirt substrate distribution relationship, and can incorporate estimates for the growth yield and maintenance, endogenous respiration or maximum substrate utilization rates, the latter can be estimated from maximum electron-transfer rates. Later, approaches to estimate substrate affinities have been proposed [41,42]. iDynoMiCS 2.0’s user defined arithmetic expressions enable specification of all these models directly in an input file rather than requiring programming. An individual-based model using thermodynamics-based yield and maintenance calculations has been implemented by Gogulancea *et al*. [43].

Positive stoichiometry signifies production, and negative stoichiometry consumption, when the reaction proceeds in the forward direction (has a positive rate). Thus, the production rate of a solute, *i*, by reaction, *j*, is given by:

$$q_{i,j}=N_{i,j}r_{j}$$ (6)

where *N* denotes stoichiometry and *r* denotes reaction rate.

#### Solutes in well-mixed environments

In compartments assumed to be well-mixed, there is neither spatial structure for solutes nor for agents, thus the rate of change is an ordinary differential equation summing inputs, outputs and reactions.

$$\frac{d}{dt}S_{s}(t)=\sum_{i\in inflows}D_{i}S_{s,i}(t)-\sum_{i\in outflows}D_{i}S_{s}(t)+q_{s}(t)$$ (7)

where *S*_*s*_*(t)* is the concentration of solute s at time *t* and *D*_*i*_ the dilution rate for a given inflow/outflow. The production rate expression *q*_*s*_*(t)* combines environmental reactions, *q*_*s*,*env*_, with reactions catalyzed by each agent, *q*_*s*,*agent*_.

$$q_{s}(t)=q_{s,env}(S(t))+q(S(t),agents(t))$$ (8)

where *S(t)* is the local solute concentration.

#### Diffusion-Reaction of solutes

Within spatially explicit compartments, the dynamics of solutes are governed by two processes: Fickian diffusion and reactions.

For each solute, the rate of change for each solute is now given by the elliptic Partial Differential Equation (PDE) that combines Fickian diffusion, given by the divergence div of the diffusional flux driven by the concentration gradient ∇*S*_*s*_, and reaction [44].

$$\frac{\partial }{\partial t}S_{s}(x,t)=div(\omega_{s}(x)∙\nabla S_{s}(x,t))+q_{s}(x,t)$$ (9)

where ***x*** is the spatial position, ω_*s*_ is the local diffusivity, and *S*_*s*_*(****x***,*t)* the local concentration of solute *s*. Dynamics at compartment edges are governed by boundary conditions.

As in iDynoMiCS 1 [17] and other biofilm models, a pseudo steady-state assumption is made to model solute dynamics because reaction and diffusion processes are several orders of magnitude faster than biomass growth, decay and detachment processes [45]. Hence, reaction and diffusion rates rapidly reach a pseudo steady-state while the biomass distribution is changing so slowly that it can be considered ‘frozen’ [46]. This time scale separation drastically reduces the computational demand of the simulation but it should be checked whether the pseudo steady-state assumption is reasonable.

#### Boundaries

Spatially explicit compartment boundaries can be of different types. (a) Solid boundaries where neither agents nor solutes can pass through (Neumann or no flux boundary condition). (b) Fixed concentration or Dirichlet boundary conditions where the solute concentrations are fixed to preset values or determined dynamically by an ODE such as Eq 7 for a connected well-mixed compartment. (c) Periodic boundaries where agent and solutes can move through the boundary to the opposite side of the domain, which ensures identical concentrations on each side to avoid edge effects.

Well-mixed compartments may also exchange solutes and agents with other well-mixed or spatially explicit compartments through its boundaries. (d) A boundary connecting a spatially explicit with a well-mixed compartment, where solute concentration gradients in the spatially explicit compartment result in a diffusive flux through the boundary determined by the concentration gradient at the boundary. (e) An inflow boundary with preset solute concentrations and flow rate. (f) An outflow boundary with the concentrations matching the content of the well-mixed compartment and a preset flow rate, which may match the inflow. The well-mixed compartment may change volume over time if inflow and outflow rates do not match. An outflow boundary can be set to retain the agents present in the well-mixed compartment to model biomass retention in a retentostat.

#### Plasmid dynamics

Plasmid dynamics incorporates conjugative transfer and segregative loss of plasmids. Plasmids are included as aspects of an agent, with loss defined as an event occurring upon agent division. The dynamics of conjugation modeled here follow the known behavior of F-pili driven plasmid transfer. The dynamics of transfer were based on the live cell imaging by Clarke *et al*. (2008) [47]. A detailed description is included in S1.11.

## Results

We start by summarizing the strategy and results of our model verification efforts that were focused on comparing our numerical solvers against analytical solutions in the simpler test cases and well tested solvers implemented in other software in the more complex test cases. This is followed by using benchmarks. First the standard Benchmark 3 for comparison of biofilm models as previously done for iDynoMiCS 1. Then a second, new benchmark that is highly sensitive to initial conditions and spatial structures due to positive feedbacks where we can compare iDynoMiCS 2.0 results with BacSim and also investigate the often-neglected effect of different biofilm spreading mechanisms. Finally, we demonstrate some of the new capabilities of iDynoMiCS 2.0 –simulating filaments which requires FbM–and show some surprising new results.

### Model verification and performance

#### Component testing and solver verification

iDynoMiCS 2.0 has undergone a rigorous verification process. We focused on numerical solvers, writing code to inspect the state at each iteration and diagnose convergence of solvers. This process helped eliminate bugs and software inefficiencies. It also demonstrated solutions were numerically correct with deviations of <0.02% in all test scenarios with known analytical solutions. The testing process included single-component testing (or unit testing), multi-component testing, stress testing and benchmarking, following a strategy of increasingly complex test scenarios where analytical solutions were known for simpler cases, numerical solutions from well-tested other solvers for intermediate cases and comparison to output of a set of other models for the most complex test cases. Single-component testing entailed the testing of individual parts of the framework against known solutions, such as biomass and solute concentrations in a chemostat [48] or predicted convergence. This included tests for collision detection and collision response (S1.2), (bio-)chemical conversion and material transport in a well-mixed compartment (S1.3), microbial growth in a well-mixed compartment (S1.3) and (bio-)chemical conversion and diffusion in a spatially explicit compartment (S1.3).

Multi-component testing where multiple parts of the framework are tested simultaneously was performed through using various test scenarios. The goal of these tests was to see whether multiple components worked correctly in unison and whether iDynoMiCS 2.0 can perform more complex scenarios stably and reproducibly. Test scenarios are listed in S1.9, protocol files for all scenarios are available on the iDynoMiCS 2.0 GitHub repository.

#### Stress testing

**Fig 3:**
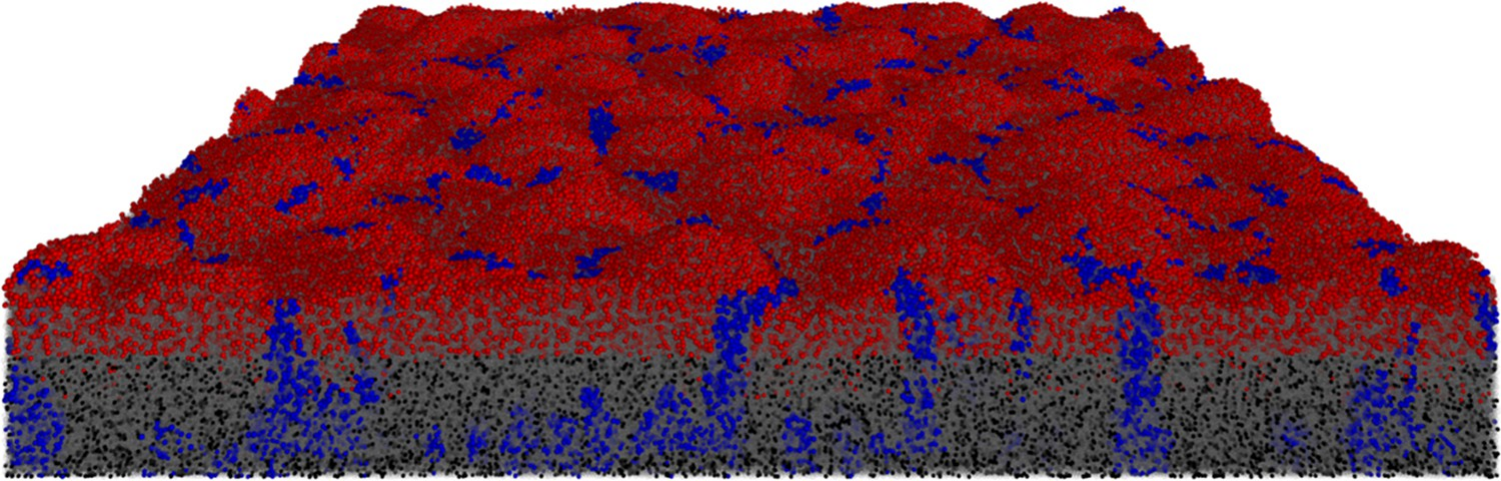
iDynoMiCS 2.0 was capable of simulating large 3D biofilms. A nitrifying biofilm was initiated with 1,000 Ammonium Oxidizing Organisms (red) and 1,000 Nitrite Oxidizing Organisms (blue) in a 500x500x500 μm domain. Growth kinetics were adopted from Hubaux et al. [49]. Both species produced EPS particles (gray semi-transparent). Agents that dropped below 20% of their division mass as a result of endogenous respiration (maintenance metabolism) became inactive (black). The 175-day biofilm contained 1.02×10^7^ agents (bacteria and EPS particles).

Stress testing built on multi-component testing, but tested scalability of performance and pushed the limits of domain size. In the process, software limitations and bottlenecks were removed. This included detailed analysis of memory usage and CPU time using the Java profiler and selecting or designing efficient algorithms for specific tasks. A few impactful design choices were: The quad/octree implementation which minimizes the number of agent collision evaluations that need to be performed. The FbM algorithm, which effectively halves the number of ODEs that need to be evaluated compared to classical mechanics. Finally, making use of the pseudo steady-state assumption allows for the close approximation of solute concentrations and reaction rates with a steady-state solver. The solver automatically stops when residual errors diminish. The stress test verified that iDynoMiCS 2.0 was capable of simulating the development of a 3D biofilm over 175 simulated days. It reached >10 million agents (Fig 3, detailed description in S1.4) in 11.34 days on a single processor. Apart from the efficiency of algorithms, the complexity and parameters of the model system can affect runtime, and thus not every model implementation will be as performant.

#### Benchmarking against various types of biofilm models

**Fig 4:**
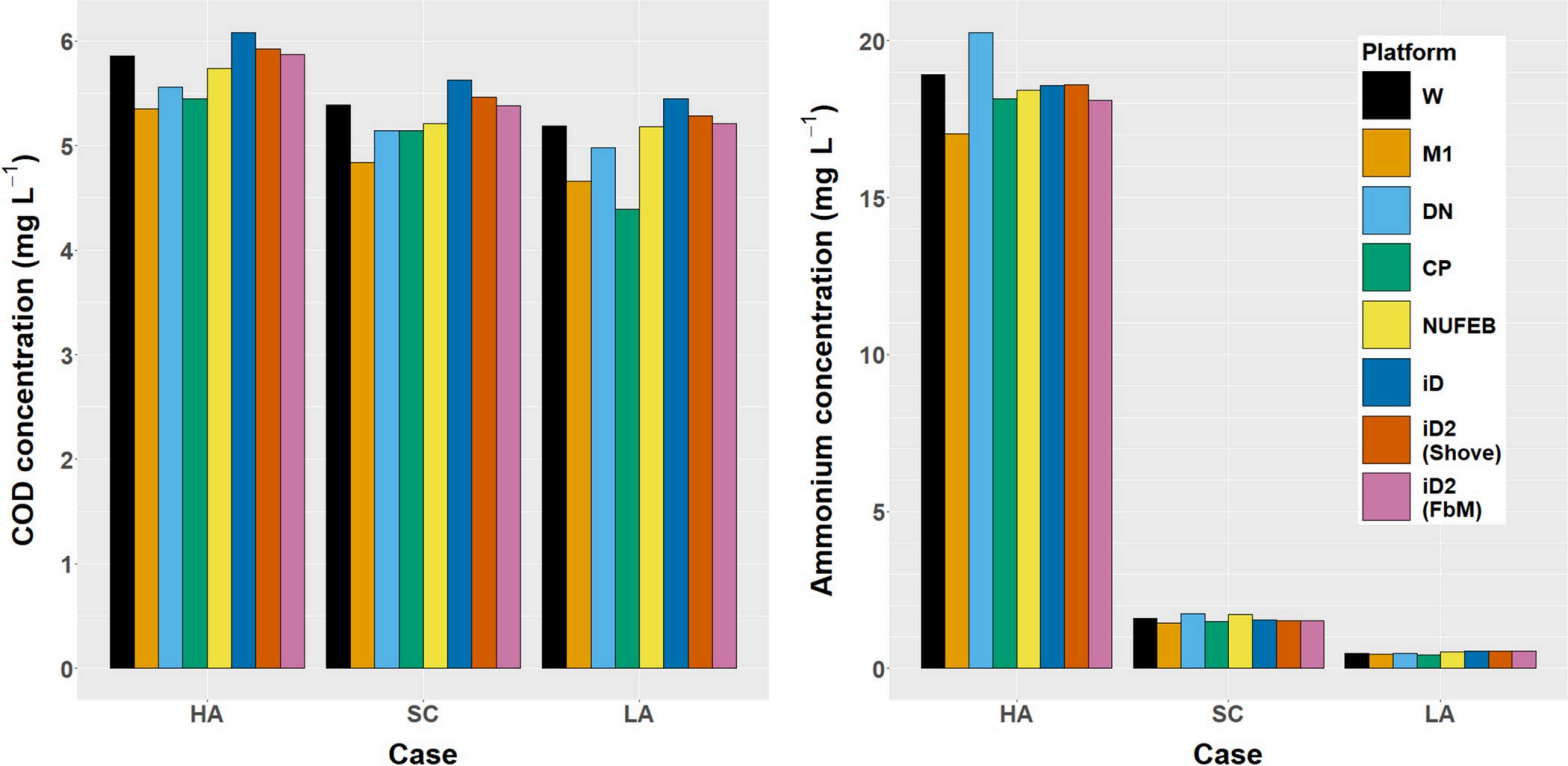
Comparing steady states in BM3. Steady state organic carbon (Chemical Oxygen Demand, COD) and ammonium concentrations in the bulk liquid for the three different BM3 cases (HA: High ammonium, SC: Standard case, LA: Low ammonium) across 7 model implementations (W: a one-dimensional continuum biomass model run on the AQUASIM software [53] and developed by Peter Reichert and Oskar Wanner [54,55], M1: a variant of the W model with a fixed boundary-layer thickness by Eberhard Morgenroth *et al*. [56], DN: a two-dimensional cellular automaton model developed by Daniel Noguera and colleagues [57], CP: a two-dimensional individual-based model, with biomass spreading via shoving, developed by Cristian Picioreanu and colleagues [58], NUFEB: a three-dimensional individual-based model by Li *et al*. [21], iD: an individual-based model by Lardon *et al*. [17] (iDynoMiCS 1), iD2: iDynoMiCS 2.0, either with shoving algorithm similar to iD or the new Force-based Mechanics). Data and analysis are included in Table I in S1 Text.

iDynoMiCS 2.0 was benchmarked against 1D or 2D continuum models, 2D Cellular Automata and 2D particle-based models including iDynoMiCS 1, using the multi-species nitrifying biofilm Benchmark Problem 3 or BM3 [50,51]. BM3 was the most complex benchmark developed by the International Water Association biofilm modeling task group to compare computational modeling approaches for biofilms and provide guidance for researchers. All models implemented the same processes. Unfortunately, some published BM3 results are limited to steady state concentrations of organic carbon (expressed as Chemical Oxygen Demand, COD) and ammonium in the bulk liquid that exchanges with the biofilm so we could only show that these results from iDynoMiCS 2.0 did not differ significantly from the distribution of results from the other models (Fig 4 and Table I in S1 Text). iDynoMiCS 1 was previously shown to produce similar results to another particle-based model, NUFEB [21,52]. We also tested and confirmed that the physically realistic biomass spreading mechanism FbM in iDynoMiCS 2.0 gave similar results to the biomass spreading by shoving in iDynoMiCS 1, and for that purpose implemented shoving in iDynoMiCS 2.0 as well (Fig 4). Since FbM, without explicitly modeled EPS, produces denser biofilms than shoving, the FbM simulations used a lower agent density than the shoving simulations such that the overall biofilm densities matched published models (see Table F in S1 Text). The biofilm density is an emergent property, which can be affected by solver parameters such as the shoving factor for shoving algorithms or the maximum permitted agent overlap and the maximum residual force for FbM. Also packing efficiency and agent sizes can play a role. The modeler should therefore always verify whether the emergent biofilm density matches the target system and adjust the model if necessary. An extensive BM3 description and results analysis is included in S1.5.

### Comparing the effect of different biomass spreading mechanisms: Biofilms promote altruism case study

**Fig 5:**
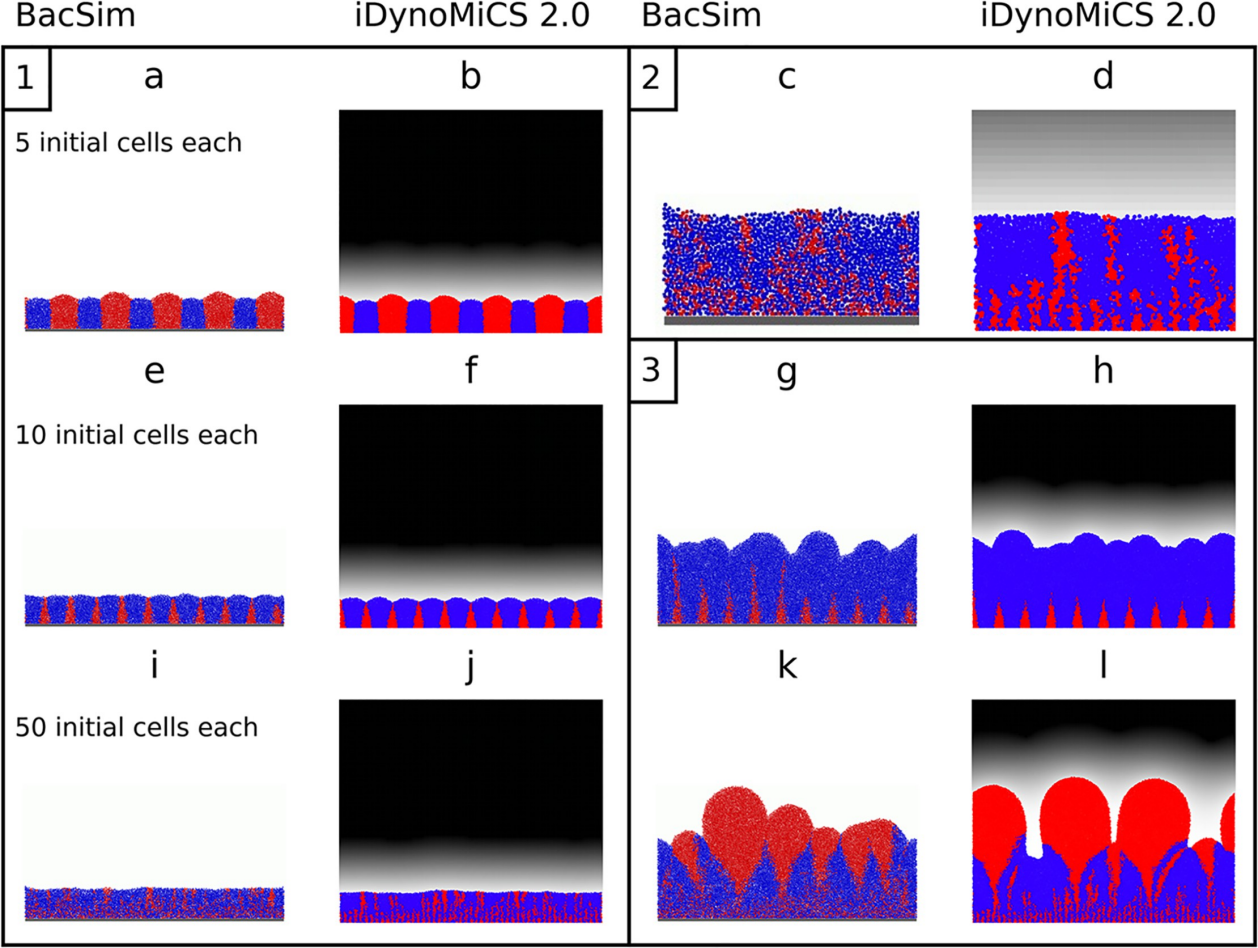
Biofilms promote altruism case study. Rate Strategist (RS, blue) and Yield Strategist (YS, red) competitions using the shoving algorithm in BacSim [59] (reproduced from “Kreft J-U (2004). Biofilms promote altruism. Microbiology 150: 2751–2760” with permission) were replicated in iDynoMiCS 2.0 with its force-based mechanics. Cells were initially placed in alternating, equidistant positions with increasing density from 5 cells per strategy (Scenario 1: a-b), 10 cells each (Scenario 2: e-h) to 50 cells each (Scenario 3: i-l and c-d). iDynoMiCS 2.0 panels show local oxygen concentration as a linear gray-level gradient from zero oxygen (0 mg L^-1^, white) to a maximum concentration (S_ox_bulk_ = 1 mg L^-1^, black). Block 1 shows 3-week-old biofilms. Block 2 zooms into panels i and j. Block 3 shows 10-week-old biofilms developed from the 3-week-old biofilms shown in the same position on the left.

As a second and new benchmark for model testing and comparison, we have chosen a biofilm scenario where a positive feedback can amplify initially small differences leading to divergent results. This was thus a good opportunity to examine the effect of different biomass spreading mechanisms, comparing BacSim, the first implementation of shoving for biofilms [59] with iDynoMiCS 2.0. The scenario consisted of competing two growth strategies, a Rate Strategist (RS) that grew faster at every substrate concentration (higher *μ*_*max*_, same specific affinity, i.e., initial slope of the Monod kinetics) but had a lower growth yield, with a Yield Strategist (YS) that grew more slowly but had a higher growth yield and therefore converted the substrate diffusing into the biofilm with higher efficiency into biomass. This more economical use of resources is an example of altruistic behavior as it benefits selfish neighbors more than self [59]. Model parameters are in Table K in S1 Text. We found that both IbMs generated the same qualitative outcomes, where YS won at lower initial density (Fig 5A and 5B), RS won at intermediate initial density (Fig 5E–5H) and at even higher initial density, YS won again due to clusters of YS ending up on the biofilm surface by chance and then growing better as clusters of cells which are competing less with each other (Fig 5I–5L). This combination of chance events–formation of a small cluster of YS cells at the biofilm surface–with positive feedback became obvious after longer simulations (Fig 5K and 5L).

While the qualitative outcomes were the same, the initial density thresholds separating regions where different strategies win were somewhat shifted. This was a result of the different biomass spreading algorithms: the shoving in BacSim led to more open spaces and increased mixing of cells locally, compared to force-based mechanical relaxation in iDynoMiCS 2.0, which in this case without EPS production only avoided overlap between agents but did not push cells further away (Fig F in S1 Text). Note this resulted in increased overall biofilm density in iDynoMiCS 2.0. To compensate for this effect, we had to reduce agent density in iDynoMiCS 2.0 using FbM (Table K in S1 Text) to maintain similar biofilm densities. Shoving can implicitly model the effect of EPS production generating space between agents, while EPS particles need to be included explicitly to generate space when using FbM simulations. iDynoMiCS 2.0 can do both.

### Filaments outcompeted cocci regardless of growth strategy and RS filaments expanded faster

**Fig 6:**
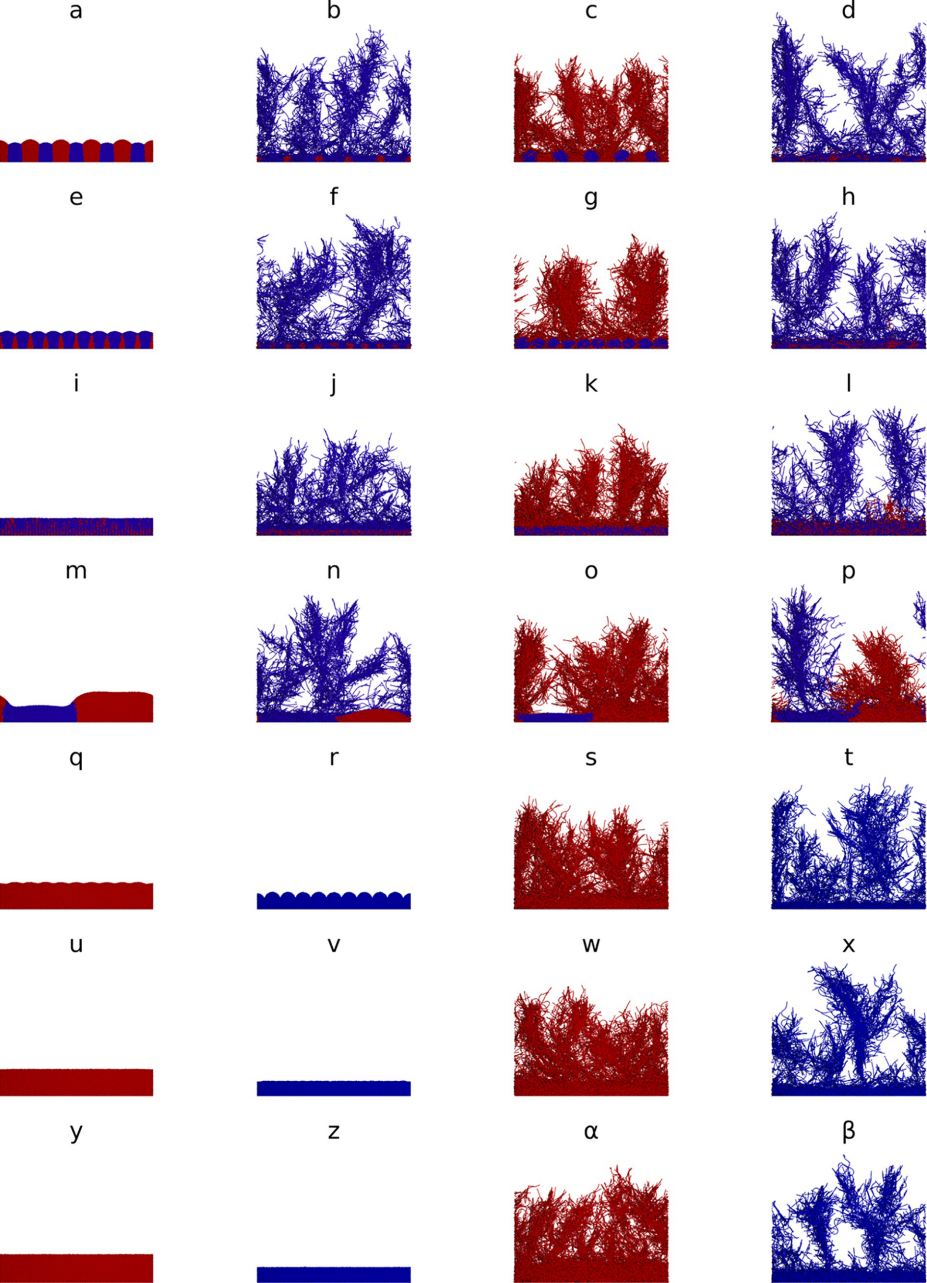
Filaments rule and gave Rate Strategists an advantage. Rate Strategists (RS, blue) and Yield Strategists (YS, red) competed in a 3D biofilm domain (200x200x12.5 μm) for 3 weeks. In the first 4 rows, different strategies competed. Column 1 corresponds to spherical cell scenarios in Fig 2 of Ref [59] but were now simulated in 3D. In column 2, RS formed filaments and in column 3, YS formed filaments. Filaments won regardless of strategy. In column 4, both formed filaments and RS won or likely won. The last 3 rows show single species ‘controls’ with 10, 20 or 100 initial agents. The first two columns show simulations with spherical YS or RS agents while the last two columns show filament forming YS or RS agents. See Fig 7 for corresponding time courses. Duplicate simulations are shown in Fig G in S1 Text. The filamentous microbes incorporate a basic life cycle in which initially spherical agents extend into rod shaped agents to further extend into multi-segmented filaments as described in S1.14.

**Fig 7:**
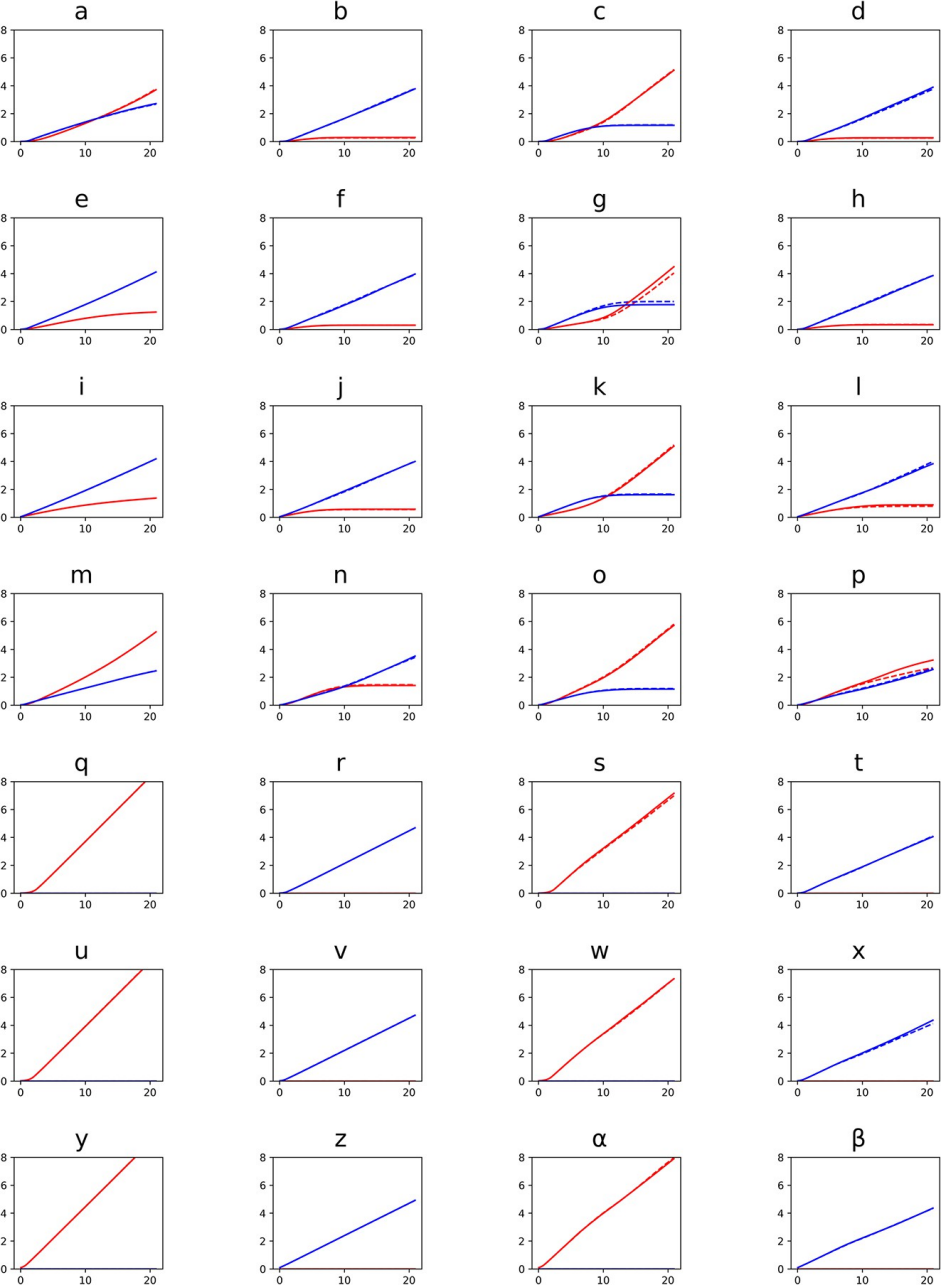
Growth curves corresponding to competitions of Rate Strategists (RS, blue) and Yield Strategists (YS, red) in Fig 6. Duplicates are plotted with dashed lines. Divergence between replicates is most visible in panels g and p. In panel p, it is too early to definitely call the outcome of competition, but it is likely that RS would win given the biofilm structure after 3 weeks (Fig 6P).

Building on the biofilms promote altruism case study and the capability of iDynoMiCS 2.0 to simulate spherical, rod-shaped and filamentous microbes, we asked whether filamentous growth provides an additional advantage to Yield Strategists (YS). Since sufficiently large clusters of the cooperative YS cells outcompeted the Rate Strategists (RS) (Fig 5), we reasoned that growing as a filament, which can be considered to be a cluster of cells in one dimension, would give YS an additional advantage. Hence, we competed all combinations in a biofilm setting: coccoid RS vs. coccoid YS, filamentous RS vs coccoid YS, coccoid RS vs. filamentous YS and filamentous RS vs. filamentous YS. As filaments need a larger domain and freedom to bend and spread in all directions, these simulations required a 3D domain of sufficient size. The z-dimension was expanded to 12.5 μm, whilst keeping solute resolution at 1.5625 μm. Since the shoving algorithm cannot properly deal with filaments, the FbM of iDynoMiCS 2.0 was required. It turned out that filaments were superior to cocci regardless of growth strategy–because filaments quickly gained access to the higher substrate concentrations at the top of the domain where the source of the substrate was located (Fig 6). This range expansion strategy of filaments is similar to cells producing EPS to rise quickly above biofilm neighbors towards the substrate source above, or trees growing faster to the top of the canopy to gain better access to sunlight [60], or the foraging strategy of cord-forming fungi that can form ‘bridges’ between discrete and sparsely scattered patches of nutrient resources [61]. Surprisingly, RS filaments won the competition against YS filaments in each case where the final outcome can be inferred (Fig 6 shows biofilm structures and Fig 7 shows population dynamics over time). A striking difference between Rate Strategist filaments and Yield Strategist filaments is the more open and less dense ‘forest’ structure produced by Rate Strategists. We suggest that the lower substrate consumption rate of the Yield Strategists allows their filaments to grow better in deeper regions of the biofilms than the Rate Strategist filaments, which gain a larger advantage when they happen to grow towards the top. Thus, the stronger competition (or self-inhibition) between Rate Strategist filaments favors expansion over density, leading to a ‘fluffier forest’ structure.

## Discussion

Individual-based models in microbial ecology are uniquely capable of capturing local interactions, individual heterogeneity and adaptation, stochastic processes and emergent properties of biofilms or other spatially structured assemblages [62–71]. The number of publications utilizing IbMs in this field has rapidly increased since the 1990s (S1.13 Fig I in S1 Text), due to an increased recognition of its potential and facilitated by an increased availability of ready-to-use IbM platforms (discussed below). From its inception, the goal of iDynoMiCS has always been to provide a well-tested, general-purpose platform for individual-based microbial community modeling, enabling users to specify their model in a structured text file rather than requiring them to program, thereby aiding microbial ecology and synthetic biology research, which seeks to reach a mechanistic and predictive understanding of the interactions of natural or synthetic microbes in the environment. The environment for microbes includes engineered reactors and buildings as well as plant and animal hosts. Here, we present iDynoMiCS 2.0, which has been rewritten from scratch to enable flexible agent and process specifications using orthogonal modules called “aspects”, thus removing key limitations of the original iDynoMiCS 1. In addition, we expanded the functionality, most importantly a force-based mechanical interaction framework enabling new agent morphologies such as rods and filaments, a new flexible approach to (bio-)chemical conversions enabling any type of kinetic expression, improvements that make the software easier to use including a GUI, a new protocol and program structure, unit conversions and a large number of efficiency improvements allowing for large scale (10+ million agents) simulations. We have further subjected iDynoMiCS 2.0 to rigorous testing to ensure that the platform and its individual components function correctly. In addition to standard unit tests, we have verified the accuracy of its numerical solvers by using a series of increasingly complex test cases from simpler ones with analytical solutions to more complex ones that have to be compared to independent solvers, culminating in the established Benchmark 3 comparison of nitrifying biofilms with different biofilm modeling approaches and a comparison with prior BacSim simulations of the biofilms promote altruism test case because its positive feedback in the growth of cooperative cell clusters results in higher sensitivity to local cluster formation in biofilms.

Biomass spreading mechanisms can affect model outcomes, especially when mixing of different species favors a species that can take advantage of becoming embedded in a cluster of cells of the other species, e.g., if the latter is more rapidly growing towards an energy source. Using nitrifying biofilms, it was previously shown that Cellular Automata (CA) cell division rules led to larger scale stochastic mixing of nitrite oxidizers into the (under specific conditions) more rapidly expanding incomplete ammonia oxidizers than a shoving algorithm that minimized cell-cell overlap; shoving percolated expansion through the biofilm leading to limited, localized mixing. This resulted in a substantially higher fitness of the nitrite oxidizers in the CA simulations as some of them were ‘going with the flow’ of the ammonia oxidizers towards the oxygen supply [15]. Here, we compared a shoving algorithm with FbM using the “biofilms promote altruism” case study. FbM led to even more limited and localized mixing, producing sharper boundaries between different clusters than shoving (Fig 5). As a result, the chance of clusters of yield strategists to emerge out of clusters of rate strategies (after the latter had overgrown them) and thus eventually winning the competition, was reduced. The biofilm structures resulting from FbM are reminiscent of the smooth, gradual boundaries of biomasses in continuum models of biofilms where biomass spreading is proportional to the pressure gradient [72] (Darcy’s law), but the mixing is more limited with the FbM. Note that continuum models where biomass spreading is driven by density-dependent diffusion can lead to complete mixing if counter-diffusion is not considered and gradual but large-scale mixing if it is [73]. Combining our new results with Kreft *et al*. (2001) [15], we can rank biomass spreading mechanisms in order of increasing and larger-scale mixing: FbM < shoving < CA. The importance of even subtle differences in biomass spreading mechanisms for biofilm pattern formation and population fitness should become more widely recognized as model predictions can be substantially affected. Although FbM allows for a highly detailed description of interaction forces, the question whether this accurately describes the interactions and behaviors of microbes in a biofilm remains open. Advanced experimental techniques, such as Atomic Force Microscopy (AFM) as applied by Kang and Elimelch [74] and high-resolution microscopy coupled with particle tracking as applied by Rogers *et al*. [75], can be used to obtain detailed insight into the physical interactions on an individual cell level and enable the observation of cell motion within biofilms. These methods could be used for model validation.

Morphology matters. There is a great variety of shapes [76] and sizes [77] of microbes while microbes of the same species usually have similar shape and size. Young [78] argued that the variety and uniformity of microbial morphology, the ability of bacteria to actively modify their shapes based on internal or external cues and evolutionary selection towards specific shapes all suggest that bacterial morphology is as important as other traits. Different morphologies may be selected for by different selective pressures and ecological niches such as nutrient limitation, predation, attachment, passive dispersal, active motility and differentiation [78,79]. IbMs have previously been utilized to demonstrate how cell shape can play an important role in spatial organization and evolutionary fitness in biofilms [80,81]. Sphere-shaped, rod-shaped and filamentous microbes are commonly found and can already be modeled with iDynoMiCS 2.0 and the implemented ball-spring approach facilitates future extensions to branching filaments or other morphologies.

Filaments win. Our filament case study utilized FbM to simulate filaments consisting of sphere-shaped agents and demonstrated that filamentous growth can provide a strong competitive advantage under nutrient limiting conditions (Fig 6). The advantage derives from the ability of filaments to focus the growth of biomass into one direction rather than merely producing offspring adding to an existing heap of cells. This way, longer distances can be covered and new, nutrient rich territories colonized. This is similar to the strategy of cord-forming fungi who can quickly grow towards new resource hotspots [61,82] and microbes that push themselves towards the nutrient source by producing copious amounts of low-density EPS [60]. The advantage of clusters of yield strategists, who compete less and grow faster than a cluster of rate strategists (of the same size and at the same flux of resources into the cluster), turned into a disadvantage as rate strategists who have reached the top of the biofilm where substrate flux was highest experienced stronger positive feedback than yield strategists. This suggests that filamentous growth is a strategy to escape competition between siblings. Given the huge advantage of filamentous growth found here, the question why filamentous growth is not more common in bacteria arises. It is certainly common in fungi and in the ecologically similar streptomycete bacteria, probably because of improved foraging for scattered patches of resources separated by terrain low in suitable resources like oases in a desert. Also in stream biofilms, filament or chain formation as employed by *Diatoma* spp. enhances nutrient access [29]. Gradient microbes such as *Beggiatoa* spp. or the intriguing cable bacteria [83] form filaments because they need to access electrons from a reduced sediment and electron acceptors from the oxidized water layer above the sediment. Filamentous bacteria are also found in activated sludge flocs in wastewater treatment, where they have the advantage of growing out of the floc into the nutrient richer bulk liquid but are selected against at the settling stage where only fast sinking sludge flocs are recirculated into the activated sludge stage [84,85]. But what are the disadvantages of filaments? Depending on the environment, several disadvantages may arise. Filaments are not only more exposed to beneficial resources but also mechanical or physiological stresses and attack by phages or predators, although some predators may be deterred from grazing larger cells [78]. Further, packaging biomass into smaller propagules is advantageous for dispersal. Filaments also forsake the advantages of motility [78,86]. Cell size is also an important factor for pathogenesis, some bacteria may avoid forming filaments to prevent being killed by the host [79].

iDynoMiCS 2.0 is joining an increasing number of individual-based modeling platforms that focus on microbes and can support a range of specific models. These platforms can be roughly divided into two groups, based on their subcellular versus ecological dynamics origin and focus. The former group of platforms comes from systems and synthetic biology and seek to discover how specific microbial community behaviors or phenomena can be achieved through the creation of synthetic microbial communities [87]: CellModeller [88], BSim 2.0 [89] and gro [90]. They can simulate microbial communities made up of rod-shaped microbial agents with specific metabolic, sensing and signaling properties. All three can simulate gene regulatory networks and diffusion of signaling molecules in order to explore and/or design synthetic microbial communities. While gro can only simulate 2D systems, CellModeller can simulate both 2D and 3D systems, while BSim 2.0 can only simulate 3D systems. In models that use these platforms, growth kinetics are typically less important than gene regulation, hence growth is modeled as a simple rate, as in CellModeller, or a rate based on cell length, as in BSim 2.0. However, gro allows growth to be based on Monod kinetics. CellModeller and gro do not include environmental constraints such as physical boundaries, thus simulated microbes tend to grow outwards to form round colonies. BSim 2.0, however, can model physical spaces such as microfluidic chemostats where cells may, e.g., grow and release diffusing signaling molecules.

The latter group of platforms originate from larger scale microbial ecology models, primarily for biofilms, which seek to explore population dynamics and ecological and evolutionary processes in biofilms. These include iDynoMiCS [17], Biocellion [91], Simbiotics [92], BacArena [93], NUFEB [21], ACBM [94] and McComedy [23]. They focus on microbial growth and metabolism and mass transport such as diffusion of solutes in order to assess how different growth strategies or metabolic interactions affect the fitness of species growing in a biofilm or impact systems-level outcomes in wastewater treatment systems or bioreactors. iDynoMiCS, NUFEB and Simbiotics can all model growth using equations originating from enzyme kinetics that determine reaction rates from substrate concentrations, such as Monod kinetics. Reaction rates and diffusion are coupled and solved using partial differential equation (PDE) solvers. These solvers are made efficient by taking advantage of a separation of timescales, where, e.g., growth of microbes is on a much slower timescale than diffusion. iDynoMiCS and NUFEB both simulate a diffusion boundary layer—a region around the biofilm in which diffusion dominates the transport of solutes—as distinct from the rest of the spatial domain which is well-mixed.

BacArena and ACBM are unique in utilizing flux-balance analysis to estimate the metabolic flux through ‘individual’ grid elements, based on their local solute concentrations. Diffusion is then solved using a partial differential equation (PDE) solver. NUFEB can model agent growth based on thermodynamics, calculating the Gibb’s free energy of catabolism [43], which could also be done with iDynoMiCS 2.0 as users can specify any arithmetic function for reaction kinetics. Since BacArena and ACBM have to solve flux-balances, which is computationally demanding, the platforms are more restrictive in terms of model scale. BacArena only models agents in a fixed 2D grid, with one agent per grid cell, like a CA, while ACBM groups agents together when evaluating internal processes. The other biofilm modeling platforms simulate grid-free agents that evaluate internal processes on an individual basis. Agents can excrete small particles representing EPS. NUFEB, Simbiotics and iDynoMiCS 2.0 also allow adhesive forces to be modeled. In NUFEB and iDynoMiCS 1, agents are spherical, while in Simbiotics, ACBM or iDynoMiCS 2.0, they can be spherical or rod-shaped.

Some of the models can simulate fluid motion or advection in addition to diffusion. CellModeller implements an implicit advection model which imposes a linear bulk flow in a given direction. NUFEB can simulate computationally demanding fluid dynamics explicitly through coupling with the fluid dynamics toolbox OpenFOAM, which can solve the fluid velocities based on the biofilm geometry (one way coupling). Forces are then applied to agents based on these flow velocities.

The suitability of different individual-based modeling platforms depends on the needs of the user. For exploring synthetic bacterial communities where gene regulation and signaling circuits are engineered into cells, CellModeller, gro or BSim 2.0 may be the most suitable platforms. When details of intracellular dynamics are less relevant or simply unknown and the focus is on interactions between agents and with the environment, such as in biofilms or other spatially structured habitats where mass transport is crucial, NUFEB and iDynoMiCS 2.0 may be the most suitable systems. iDynoMiCS 2.0 offers a highly modular and customizable modeling platform, with both 2D and 3D compartments, spherical, rod-shaped and filamentous microbial agents, a sophisticated reaction-diffusion system and growth models that can be based on any kind of arithmetic expression including enzyme kinetics and thermodynamics based models. It is more straightforward to specify and implement biology in iDynoMiCS 2.0. One key drawback in comparison to NUFEB is that iDynoMiCS 2.0 currently does not model fluid dynamics or advection, and thus if these are important characteristics of the system one wishes to model, NUFEB may be more suitable. BacArena and ACBM offer flux-balance models for metabolism, but therefore come with other limitations.

For specific applications, other Agent-based or related bottom-up modeling platforms are worth considering. IndiMeSH [95] is an IbM platform capable of simulating laboratory models of soil habitats. CHASTE [96], BioDynaMo [22], PhysiCell [97] and compuCell3D [98] have been primarily used for tissue modeling, which could make them applicable to the somewhat similar biofilms. Morpheus [99], like compuCell3D, utilizes a cellular Potts model to model multicellular systems. Further, there are several general purpose AbM libraries or toolkits, which facilitate the programming of a specific model by providing a wide range of common routines so models can be specified with a high-level language tailored for this purpose. These libraries could be suitable for certain microbial community models in addition to various other fields of research. They include NetLogo [100], FLAME [101], Mason [102], Repast simphony [103] and others.

iDynoMiCS 2.0 has been developed from scratch because the hierarchical inheritance of agent features in iDynoMiCS 1 prevented the fully flexible pick and mix approach to agent characteristics and processes that was required for further expansion of capabilities. Moreover, iDynoMiCS 2.0 sports several key enhancements, such as the ability to simulate spherical, rod-shaped and filamentous microbes and using Force-based Mechanics for biomass spreading, which we show can have important consequences. It can simulate larger 3D domains due to efficient neighbor searching, a faster converging reaction-diffusion solver and numerous other performance improvements. iDynoMiCS 2.0 was designed with both ease of use, from a GUI to unit conversions, and ease of extension in mind, providing a solid and well tested simulation platform for a wide variety of microbial community studies to come. We showcased the simulation of filamentous microbes using the biofilms promote altruism case study and found that the rate strategists gained a stronger advantage from filamentous growth because their tips can escape from the stronger competition between themselves. This demonstrates just one of many new possibilities of iDynoMiCS 2.0.

## Supporting information

S1 Text Supporting information for: Is it selfish to be filamentous in biofilms? Individual-based modeling links microbial growth strategies with morphology using the new and modular iDynoMiCS 2.0. **Table A. In total, 36 collision detection scenarios were included as standard unit tests.** All tests include two objects to create one of the following scenarios: object-object overlap (hit), no overlap (miss), overlap through a periodic boundary (periodic hit) and no overlap, but proximity through a periodic boundary (periodic miss). The sphere and rod objects correspond to agent shapes. Solid boundaries utilize an (infinite) plane object to allow for agent interactions. The voxel is a cube aligned with the coordinate grid. Numbers indicate the number of different configurations tested. In all tested scenarios the collision detection algorithm correctly detected the hits and misses. **Table B. Parameters used in the numerical tests of the chemostat solver. Table C. Parameters used in the numerical tests of the spatial domain in iDynoMiCS 2.0.** The total biomass was higher for the thick layer, all other parameters were identical. **Table D. Parameters for the two-species nitrifying biofilm model.** All kinetics for this model are based on Hubaux *et al*. [2]. **Table E. Petersen (stoichiometric) matrix for reactions in the stress test. Table F. Parameters used in the Benchmark 3 simulations. Table G. Petersen (stoichiometric) matrix for reactions in the Benchmark 3 simulations**, adapted from Rittmann *et al*. [12] and Lardon *et al*. [4]. Biomasses are denoted with X. Specifically, X_H_ = heterotroph active biomass, X_N_ = nitrifier (autotroph) active biomass. Substrate concentrations are denoted with S, S_S_ for the organic substrate COD, S_N_ for ammonium and S_O2_ for oxygen. For descriptions of the other parameters, see Table H. **Table H. Kinetic parameters in the Benchmark 3 models. Table I. Steady state substrate concentrations in the various IWA task group models and in iDynoMiCS 1 and iDynoMiCS 2.0.** Results for the latter models were averaged over the stochastic steady states. Hotelling’s T^2^ tests were performed to compare the results from iDynoMiCS 2.0 to those from all other models, including the IWA models, NUFEB and iDynoMiCS 1. **Table J. Steady state areal biomass density** (mass per unit surface area) of different types of biomass in the biofilm. Hotelling’s T^2^ tests were performed to compare the results from iDynoMiCS 2.0 to those from the IWA models. Biomass density was not reported in the NUFEB model benchmark, and it is thus not included in this comparison. **Table K. Model parameters for 3D simulations of the “biofilms promote altruism” case study. Table L. A selection of test protocols that are included with iDynoMiCS 2.0. Table M. Parameters required for the plasmid dynamics process manager in iDynoMiCS 2.0. Fig A. FbM led to rapid relaxation of mechanical stress from an initially over-compressed state.** Left panels from the top showing highest interaction force next to the biofilm structure: 275.1 fN for the initial state, 8.9 fN after 100 steps, 3.6 fN after 200 steps, 0.08 fN after 1,000 steps. Panel on the right shows exponential drop of the highest interaction force towards zero, demonstrating convergence of the FbM solver. **Fig B. Results of numerical tests of the ODE and PDE solvers.** Red lines show expected steady states. (a) Results from the non-growing chemostat population. The substrate concentration asymptotically approached the expected steady state of 400 mg L^-1^. (b, c) Results from the growing chemostat population. The concentrations first overshot and then asymptotically approached the expected steady state of 498.33 mg L^-1^ for the biomass in the deterministic model (b) and a single repetition of the stochastic model (c). The expected solute concentration of 6.67 mg L^-1^ was reached in both versions of the model. The graphs are indistinguishable for the stochastic and deterministic model (d). (e) Results from the thin cell layer. The concentration at the biofilm surface matched the predicted concentration of 1.8 mg L^-1^. (f) Results from the thick cell layer. The vertical line marks the biofilm surface. The substrate concentration gradient was linear in the concentration boundary layer above the biofilm surface and then decreased towards zero at the inert boundary at height 0, as expected. **Fig C. Agent mass in the large-scale stress test simulation of a biofilm in 3D.** The autotrophic nitrifying biofilm was initiated with 1 mg Ammonium Oxidizing Organisms (AOO, red) and 1 mg Nitrite Oxidizing Organisms (NOO, blue). Both species produce EPS particles (gray). Agents that drop below 20% of their division mass as a result of endogenous respiration/decay became inactive (black). At 175 days, the biofilm contains 1.02×10^7^ agents. **Fig D. BM3-iD2 Spatial biomass distribution in the three cases using shoving.** Left column shows the average areal density of each biomass type. Error bars show the standard deviation based on the three replicates run for each case. Right column shows an example from the final timestep of one replicate for each case. Results are from the simulations with the shoving biomass spreading algorithm. Coloring of agents shows the proportion of biomass that is active (bright) or inert (dark). Time courses are shown in Fig E. **Fig E. BM3-iD2 solute concentrations and areal biomass densities over time in the three simulated cases.** Lines of different shades of the same color represent different replicates of the simulations run with different random number seeds. Three replicates were run for each case. Results are from the simulations with the shoving biomass spreading algorithm. Spatial biomass distributions are shown in Fig D. **Fig F. Comparison of agent density (pg μm**^**-3**^**) distributions in biofilms simulated in BacSim using the shoving algorithm vs iDynoMiCS 2.0 using FbM.** The panels correspond to Fig 5A (left) and B (right). The computational domain was split into 100x100 grid elements, each received the full mass of agents whose center of gravity was inside the grid element, division by the grid element volume gave the local biofilm density (pg μm^-3^). iDynoMiCS 2.0 agent density was reduced by 53% in order to achieve a similar overall biofilm density. With BacSim, the biofilm density at the base was observed to be significantly lower than in the rest of the biofilm. The BacSim simulations further showed a higher standard deviation amongst bins, due to the higher agent density in these simulations. **Fig G. Replicates of biofilms promote altruism case study simulations shown in Fig 6.** Rate Strategists (RS, blue) and Yield Strategists (YS, red) competed in a 3D biofilm domain (200x200x12.5 μm) for 3 weeks. In the first 4 rows, different strategies competed. Column 1 corresponds to spherical cell scenarios in Fig 2 of Ref [14] but were now simulated in 3D. In column 2, RS formed filaments and in column 3, YS formed filaments. Filaments won regardless of strategy. In column 4, both formed filaments and RS won or likely won. The last 3 rows show single species ‘controls’ with 10, 20 or 100 initial agents. The first two columns show simulations with spherical YS or RS agents while the last two columns show filament forming YS or RS agents. The filamentous microbes incorporate a basic life cycle in which initially spherical agents extend into rod shaped agents to further extend into a multi-segmented filaments as described in S1.14. **Fig H. Preview of the iDynoMiCS 2.0 GUI during simulation.** The GUI may be used to review, edit or create protocol files before running them. During the simulation, the simulation state may be viewed but no longer edited (left). The GUI can further provide useful feedback including key information such as substrate concentration, species abundance, convergence of the reaction diffusion solver, etc. through the console. Spatial compartments can be rendered directly to visualize agent distribution and concentration gradients (right). Lastly the GUI can be used to extract key data from iDynoMiCS 2.0 output files, convert between EXI and XML files and convert numbers between different unit systems including SI and iDynoMiCS 2.0 base units. **Fig I. The number of publications of microbial IbM on PubMed since 1995.** A simple search query on PubMed reveals a growing trend in applying IbM to microorganisms. The following query was used: “((biofilm) OR (microbial)) AND ((individual-based) OR (agent-based)) AND (model)”. **Fig J. Spherical agent division.** Resulting agents are placed in opposite directions, residual overlap is resolved with FbM or shoving. **Fig K. Agent division with rod morphology.** The two resulting agents initially take the same place as the original agent with their new mass points placed in between on opposite sides of the original agent’s center of gravity. As a result, the new agents will be compressed, which is resolved by the FbM algorithm. **Fig L. An agent that incorporates morphological shifts in its growth process.** The agent shape cycles from spherical to rod shaped and back to form two spherical agents upon division. **Box A. Example of a simple iDynoMiCS 2.0 protocol file used to specify a particular model to be read and executed by the platform. Box B. This example shows how with a few lines of code a new aspect class can be created.** In this case, it is a class that calculates a coccoid radius from its volume. Because here the abstract super class “Calculated” is extended, the newly written class integrates seamlessly in the framework as initialization and data handling is handled automatically.
(PDF)
