# Supplementary material for: Is it selfish to be filamentous in biofilms? Individual-based modeling links microbial growth strategies with morphology using the new and modular iDynoMiCS 2.0
Source: PLoS Comput Biol. 2024 Feb 29;20(2):e1011303. doi: 10.1371/journal.pcbi.1011303 (PMC10947719; doi:10.1371/journal.pcbi.1011303)
Supplement: S1 Text — Table A. In total, 36 collision detection scenarios were included as standard unit tests. All tests include two objects to create one of the following scenarios: object-object overlap (hit), no overlap (miss), overlap through a periodic boundary (periodic hit) and no overlap, but proximity through a periodic boundary (periodic miss). The sphere and rod objects correspond to agent shapes. Solid boundaries utilize an (infinite) plane object to allow for agent interactions. The voxel is a cube aligned with the coordinate grid. Numbers indicate the number of different configurations tested. In all tested scenarios the collision detection algorithm correctly detected the hits and misses. Table B. Parameters used in the numerical tests of the chemostat solver. Table C. Parameters used in the numerical tests of the spatial domain in iDynoMiCS 2.0. The total biomass was higher for the thick layer, all other parameters were identical. Table D. Parameters for the two-species nitrifying biofilm model. All kinetics for this model are based on Hubaux et al. [2]. Table E. Petersen (stoichiometric) matrix for reactions in the stress test. Table F. Parameters used in the Benchmark 3 simulations. Table G. Petersen (stoichiometric) matrix for reactions in the Benchmark 3 simulations, adapted from Rittmann et al. [12] and Lardon et al. [4]. Biomasses are denoted with X. Specifically, XH = heterotroph active biomass, XN = nitrifier (autotroph) active biomass. Substrate concentrations are denoted with S, SS for the organic substrate COD, SN for ammonium and SO2 for oxygen. For descriptions of the other parameters, see Table H. Table H. Kinetic parameters in the Benchmark 3 models. Table I. Steady state substrate concentrations in the various IWA task group models and in iDynoMiCS 1 and iDynoMiCS 2.0. Results for the latter models were averaged over the stochastic steady states. Hotelling’s T2 tests were performed to compare the results from iDynoMiCS 2.0 to those from all other models, [file pcbi.1011303.s001.pdf]

S1 Text: Supporting information for:

Is it selfish to be filamentous in biofilms? Individual-based modeling links microbial growth strategies with morphology using the new and modular iDynoMiCS 2.0

Bastiaan J R Cockx[1]*, Tim Foster[2], Robert J Clegg[2], Kieran Alden[2], Sankalp Arya[3], Dov J Stekel[3], Barth F Smets[1], Jan-Ulrich Kreft[2]*

[1]Department of Environmental and Resource Engineering, Technical University of Demark, Bygningstorvet, Bygning 115, 2800 Kgs. Lyngby, Denmark

[2]Centre for Computational Biology & Institute of Microbiology and Infection & School of Biosciences, University of Birmingham, Edgbaston, Birmingham, B15 2TT, UK

[3]School of Biosciences, University of Nottingham, Sutton Bonington Campus, Loughborough, Leicestershire, LE12 5RD, UK

*Corresponding authors: Jan-Ulrich Kreft j.kreft@bham.ac.uk, Bastiaan J R Cockx: baco@dtu.dk

Published in PLoS Computational Biology 2024: https://dx.doi.org/10.1371/journal.pcbi.1011303

S1.1 Introduction

The supplementary materials provide additional details on the iDynoMiCS 2.0 platform and the model implementations presented in the main manuscript. This includes a further description of the framework and detailed descriptions of the case studies with their parameters. Moreover, the model verification and benchmarking against prior work is presented.

S1.2 Detailed description of Force-based Mechanics (FbM) and testing

The force-based mechanical interactions between agents and agents and surfaces in iDynoMiCS 2.0 rely on both correct detection of overlapping agents or collisions and correct responses. Detection is simple for stored interactions such as the interaction between the two points of a rod cell connected by a spring or the interaction between cells in a filament also connected by springs. In this case, detection is as simple as checking whether interaction data is stored as an aspect of the agent. In the case of collisions or attractive interactions, collision detection is utilized. Different shapes as well as periodic boundaries add complexity to this routine. For verification purposes, a total of 36 collision detection scenarios (Table A) were tested and included in the software as unit-tests.

Table A. In total, 36 collision detection scenarios were included as standard unit tests. All tests include two objects to create one of the following scenarios: object-object overlap (hit), no overlap (miss), overlap through a periodic boundary (periodic hit) and no overlap, but proximity through a periodic boundary (periodic miss). The sphere and rod objects correspond to agent shapes. Solid boundaries utilize an (infinite) plane object to allow for agent interactions. The voxel is a cube aligned with the coordinate grid. Numbers indicate the number of different configurations tested. In all tested scenarios the collision detection algorithm correctly detected the hits and misses.

	Sphere + Sphere	Sphere + Rod	Rod + Rod	Plane + Sphere	Plane + rod	Voxel + Sphere	Voxel + rod
hit	1	1	1	1	1	1	6
miss	1	1	1	1	1	1	4
periodic hit	1	1	1	1	1	1	3
periodic miss	1	1	1			1	1

Correct interaction response entails relaxing mechanical stresses between agents until a relaxed state is reached. Criteria for a relaxed state can either be a threshold value for tolerated residual interaction force in the model state or a threshold value for tolerated agent overlap (µm). In the test in Fig A, an over-compressed initial state underwent 1,000 FbM iterations using its default parameters. Initial peak interaction forces dropped exponentially to asymptotically approach zero (the maximum residual interaction force, reached after 829 iterations, was less than 0.1 fN).

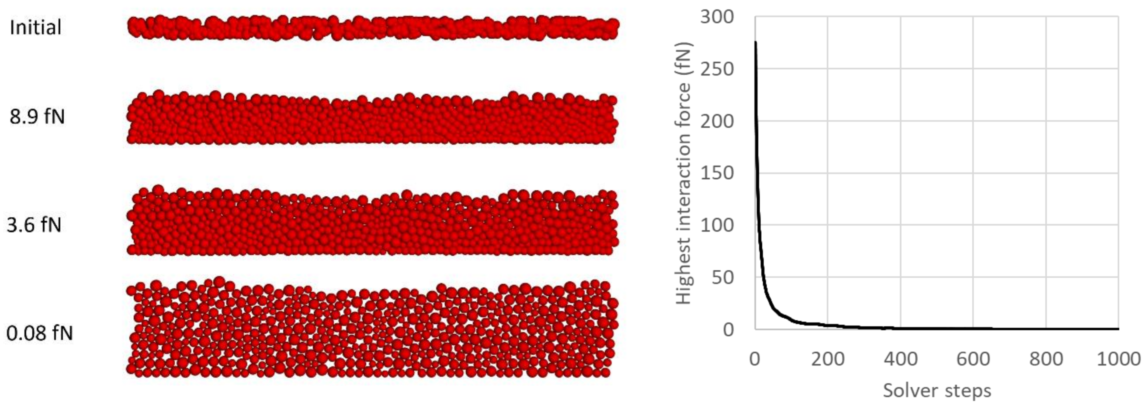

Fig A. FbM led to rapid relaxation of mechanical stress from an initially over-compressed state. Left panels from the top showing highest interaction force next to the biofilm structure: 275.1 fN for the initial state, 8.9 fN after 100 steps, 3.6 fN after 200 steps, 0.08 fN after 1,000 steps. Panel on the right shows exponential drop of the highest interaction force towards zero, demonstrating convergence of the FbM solver.

S1.3 Testing Reaction and Diffusion of Chemical Species

In iDynoMiCS 2.0, ODE and PDE solvers are responsible for modeling the diffusion and reaction (consumption or production) of solutes throughout the simulated system and are therefore responsible for the maintenance of mass balance within the model. Reactions can be chemical reactions or catalyzed by individual agents.

To test that these solvers work as intended, a range of test cases were run, which allowed the results from iDynoMiCS 2.0 to be compared with known analytical solutions. The tests were conducted starting with the simplest and proceeding to increasingly complex systems. The first two tests were non-spatial systems, used to test the ODE solver, while the latter two tests were for the more complex PDE solver in a 2D spatial system. All tests are described in full below.

1. Non-growing Catalyst Agent in a Chemostat

For this simplest test, a single agent was simulated in a chemostat compartment, consuming the inflowing solute. Fresh medium with a fixed solute concentration flowed into the chemostat at a fixed flow rate. Spent medium flowed out of the chemostat at a rate equal to the inflow. The consumption of the solute by the agent was proportional to the solute concentration and to the agent's mass. The agent was neither growing nor removed.

This system can be described by the following differential equation

$$\frac{dS}{dt} = \frac{QS_0}{V} - \frac{QS}{V} - \frac{mqS}{V} \qquad \text{(S1)}$$

Where S is the solute concentration of the substrate in the chemostat, S_0 is the solute concentration in the inflowing medium, Q is the flow rate with dimension volume per time, V is the volume of the chemostat, t is time, q is the catalyst and solute concentration specific rate of solute consumption and m is the mass of the catalyst agent.

The steady-state solution for this differential equation is

$$S^* = \frac{QS_0}{Q+mq} \qquad \text{(S2)}$$

This system was simulated in iDynoMiCS 2.0, with timesteps of 10 minutes. The steady state predicted given parameters in Table B was 0.4 g L^{-1} and the simulated concentration converged to this steady state exactly (Fig B panel a).

Table B. Parameters used in the numerical tests of the chemostat solver.

Parameter	Non-growing agent in chemostat	Growing population in chemostat
S_0	2.0 g L^{-1}	2.0 g L^{-1}
Q	0.06 pL h^{-1}	0.05 µL h^{-1}
V	1 pL	0.2 µL
q	6.0 mL g^{-1} h^{-1}	
K_S		20 mg L^{-1}
m	0.04 ng	10 ng*
μ_{max}		1.0 h^{-1}
Y		0.25 g g^{-1}

*The mass for the simulation of the growing population refers only to the initial mass.

2. Growing Population in a Chemostat

This test simulated microbial growth in a chemostat using a deterministic chemostat model and an individual-based model with stochastic agent removal. The chemostat had a constant volume with an inflowing substrate which can be converted to biomass, and an outflow removing both spent medium, and biomass (deterministic model) or agents (stochastic model), at a rate equal to the inflow. With the deterministic model, biomass removal was proportional to the dilution rate while for the individual-based model, the probability of any agent to be removed during a timestep was calculated:

$$P_{washout} = e^{\frac{Q*t}{V}} \tag{S3}$$

The agents or biomass consumed substrate and grew according to Monod kinetics:

$$\mu = \frac{\mu_{max}\, S}{K_S + S} \tag{S4}$$

Where μ is the specific growth rate, μ_{max} is the maximum specific growth rate and K_S is the half-saturation constant, the value of S at which $\mu = \mu_{max}/2$.

Here, the rate of change of substrate concentration is given by:

$$\frac{dS}{dt} = \frac{Q\, S_0}{V} - \frac{Q\, S}{V} - Y^{-1}\mu(S)P \tag{S5}$$

Where Y is the biomass yield from the substrate and P is the concentration of the biomass of all (planktonic) agents in the chemostat, with the rate of change given by:

$$\frac{dP}{dt} = -\frac{Q\, P}{V} + \mu(S)P \tag{S6}$$

This system can be solved to find the steady states for both *P* and *S*, the washout steady state of $P^* = 0$, $S^* = S_0$ [1], and the steady with agents present:

$$S^* = \frac{{}^{Q}/_{V} K_S}{\mu_{max} - {}^{Q}/_{V}} \tag{S7}$$

$$P^* = Y(S_0 - S^*) \tag{S8}$$

With the parameter values in Table B, we obtain the following steady state predictions:

$$S^* = 6.67\ mg\ L^{-1} \tag{S9}$$

$$P^* = 498.33\ mg\ L^{-1} \tag{S10}$$

Running the simulations in iDynoMiCS 2.0 yielded the expected stable steady state (Fig B panel b-d). The deterministic model yielded mean simulated values at steady state of S* = 6.67 mg L^{-1} and P* = 498.34 mg L^{-1}. These results differed from the expected steady states by 0.0008% and 0.0006%, respectively. The stochastic model naturally causes a degree of variation in the model outcomes. The average of the steady states of 9 repetitions of S* = 6.64 mg L^{-1} and P* = 498.40 mg L^{-1} was close to the same expected outcome, without any systematic deviation.

3. Thin Layer of Non-growing Cells in a Spatial Domain

In this test, a thin non-growing layer of cells, occupying one row of solver grid elements, was simulated at the bottom of a spatial compartment, with a concentration boundary layer above the cells, and a well-mixed region above that with the constant concentration of substrate S_0. Since there was no gradient of biomass or reaction rates in the horizontal direction, this is effectively a 1D system for which an analytical solution for the flux, J, can be calculated according to Fick's first law:

$$J = D\frac{dS}{dx} \quad \text{(S11)}$$

Where *J* is the areal flux density through the diffusive region, *D* is the diffusivity of the solute *S* and *x* is the vertical distance (the direction for the flux and substrate concentration gradient).

Given that at steady state, flux must be constant along the x-axis in the region where the substrate is not consumed and then starting to decline where the substrate is consumed by the cells at the bottom, we can substitute J by the areal consumption rate at the cell-layer surface. Modeling a simple consumption rate proportional to biomass and substrate concentration, we obtain:

$$\frac{m\, q\, S^*}{A} = D\,\frac{S_0 - S^*}{\Delta x} \quad \text{(S12)}$$

where S* is the steady-state concentration at the biofilm surface, A is the surface area of the biofilm and *Δx* is the depth of the diffusion-dominated boundary layer. This can be rearranged to:

$$S^* = \frac{D\, A\, S_0}{\Delta x\, m\, q + D\, A} \quad \text{(S13)}$$

Setting the parameters as shown in Table C, the predicted steady state concentration at the cell layer surface is S* = 1.8 mg L^{-1}. This was matched in the simulation (Fig B panel e). Deviations from the expected concentration are very small at each height, with the greatest deviation of 0.017% at a height of 8 µm.

Table C. Parameters used in the numerical tests of the spatial domain in iDynoMiCS 2.0. The total biomass was higher for the thick layer, all other parameters were identical.

Parameter	Thin layer	Thick layer (biofilm)
S_0	2.0 mg L^{-1}	2.0 mg L^{-1}
D	6×10^{-6} cm^2 s^{-1}	6×10^{-6} cm^2 s^{-1}
q	6 L g^{-1} h^{-1}	6 L g^{-1} h^{-1}
m	0.128 ng	1.2 ng
Δx	10 µm	10 µm
A	32 μm^2	32 μm^2

Biofilm - Thick Layer of Non-Growing Cells in a Spatial Domain

For a biofilm simulation with a thicker layer of cells following Monod kinetics, no analytical solution is available for the solute concentration at the surface of the biofilm. However, the nature of the boundary at the bottom of the domain, an inert, solid and flat surface with a no-flux (Neumann) boundary condition, provides another testable feature. As a result of the thick biofilm layer consuming substrate while it diffuses towards the bottom, the concentration gradient is expected to decrease from the maximum level in the diffusion boundary layer to become zero at the inert surface. The results of the test replicated the predicted features of the concentration gradient (Fig B panel f), suggesting that the diffusion-reaction solver and the no-flux boundary conditions in iDynoMiCS 2.0 are functioning as expected. There was no horizontal gradient or any unexpected deviations at the horizontal, periodic boundaries.

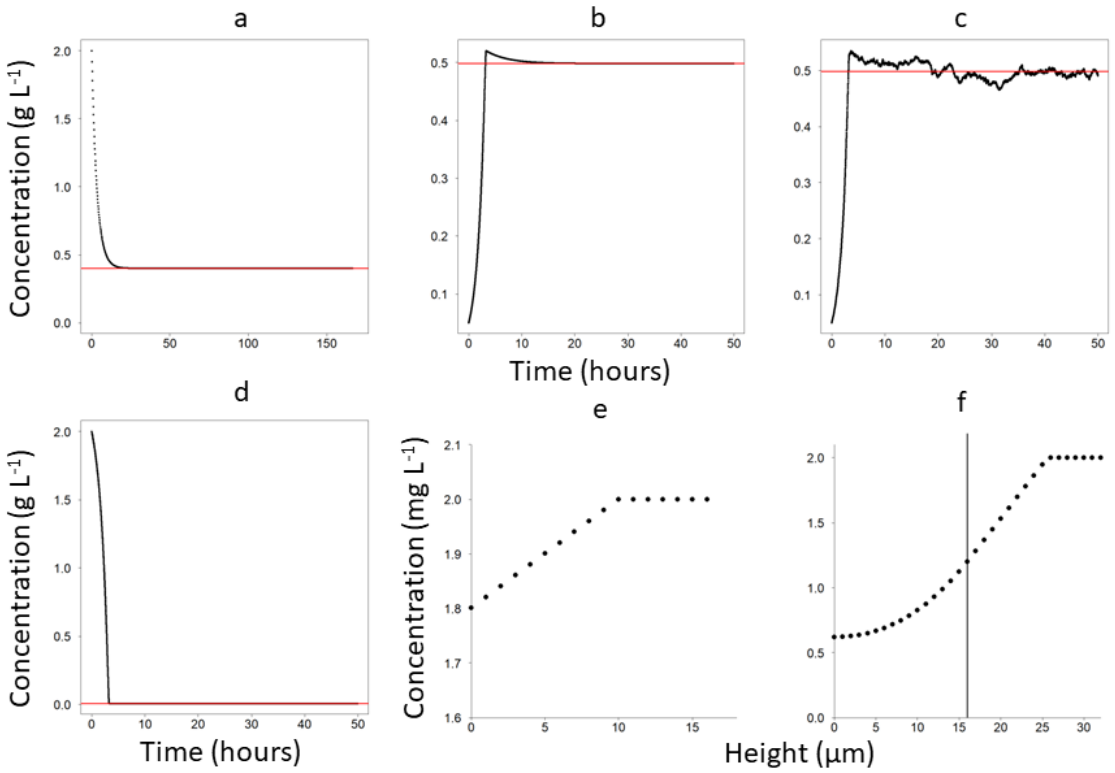

Fig B. Results of numerical tests of the ODE and PDE solvers. Red lines show expected steady states. (a) Results from the non-growing chemostat population. The substrate concentration asymptotically approached the expected steady state of 400 mg L^{-1}. (b, c) Results from the growing chemostat population. The concentrations first overshot and then asymptotically approached the expected steady state of 498.33 mg L^{-1} for the biomass in the deterministic model (b) and a single repetition of the stochastic model (c). The expected solute concentration of 6.67 mg L^{-1} was reached in both versions of the model. The graphs are indistinguishable for the stochastic and deterministic model (d). (e) Results from the thin cell layer. The concentration at the biofilm surface matched the predicted concentration of 1.8 mg L^{-1}. (f) Results from the thick cell layer. The vertical line marks the biofilm surface. The substrate concentration gradient was linear in the concentration boundary layer above the biofilm surface and then decreased towards zero at the inert boundary at height 0, as expected.

S1.4 Large scale stress-test

A two-species nitrifying biofilm model was set up to test the ability of iDynoMiCS 2.0 to simulate larger scale domains. The kinetics are based on Hubaux et al. [2]. A 500x500x500 µm spatial compartment with fixed concentrations at the top of the domain was initiated with 1,000 Ammonium Oxidizing Organisms (AOO) and 1,000 Nitrite Oxidizing Organisms (NOO), randomly distributed over the inert surface at the bottom of the spatial compartment. Model parameters are given in Table D and the stoichiometry and process kinetics are given in the Petersen matrix in Table E.

Table D. Parameters for the two-species nitrifying biofilm model. All kinetics for this model are based on Hubaux *et al.* [2].

Ammonium oxidizing organisms (AOO)			
μ_{AOO}	d^{-1}	Maximum specific growth rate of AOO	2.05
Y_{AOO}	$g_{COD}\ g_N^{-1}$	Growth yield of AOO	0.15
KNH_4,AOO	$g_{NH4\text{-}N}\ m^{-3}$	Half saturation constant for AOO	2.4
KO_2,AOO	$g_{COD}\ m^{-3}$	Half saturation constant for AOO	0.6
b_{AOO}	d^{-1}	Decay rate of AOO/End. Resp. rate	0.13
iNXB	$g_N\ g_{COD}^{-1}$	Nitrogen content in AOO	0.083
**Nitrite oxidizing organisms (NOO)**			
μ_{NOO}	d^{-1}	Maximum specific growth rate of NOO	1.45
Y_{NOO}	$g_{COD}\ g_N^{-1}$	Growth yield of NOO	0.041
KO_2,NOO	$g_{COD}\ m^{-3}$	Half saturation constant for NOO	2.2
KNO_2,NOO	$g_{NO2\text{-}N}\ m^{-3}$	Half saturation constant for NOO	5.5
b_{NOO}	d^{-1}	Decay rate of NOO	0.06
iNXB	$g_N\ g_{COD}^{-1}$	Nitrogen content in NOO	0.083

The simulation was run on a single core of an Intel Xeon E5 2660 processor with 256 GB memory, the biofilm surpassed 10 million agents after 11 days and 8 hours CPU time, less than the 171 days of simulated time of biofilm development. The simulation was stopped after 175 days of simulated time.

The AOO and NOO populations initially grew exponentially as long as growth was not limited by substrate influx and then grew linearly (Fig C) while being limited by substrate influx, until reaching a steady state after around 100 days simulated time due to the balancing of overall growth and decay rates with only minor fluctuations in population size. There was no decline as bulk concentrations were kept constant. EPS and inert agents were assumed not to decay in this model, consequently these agent populations continued to increase.

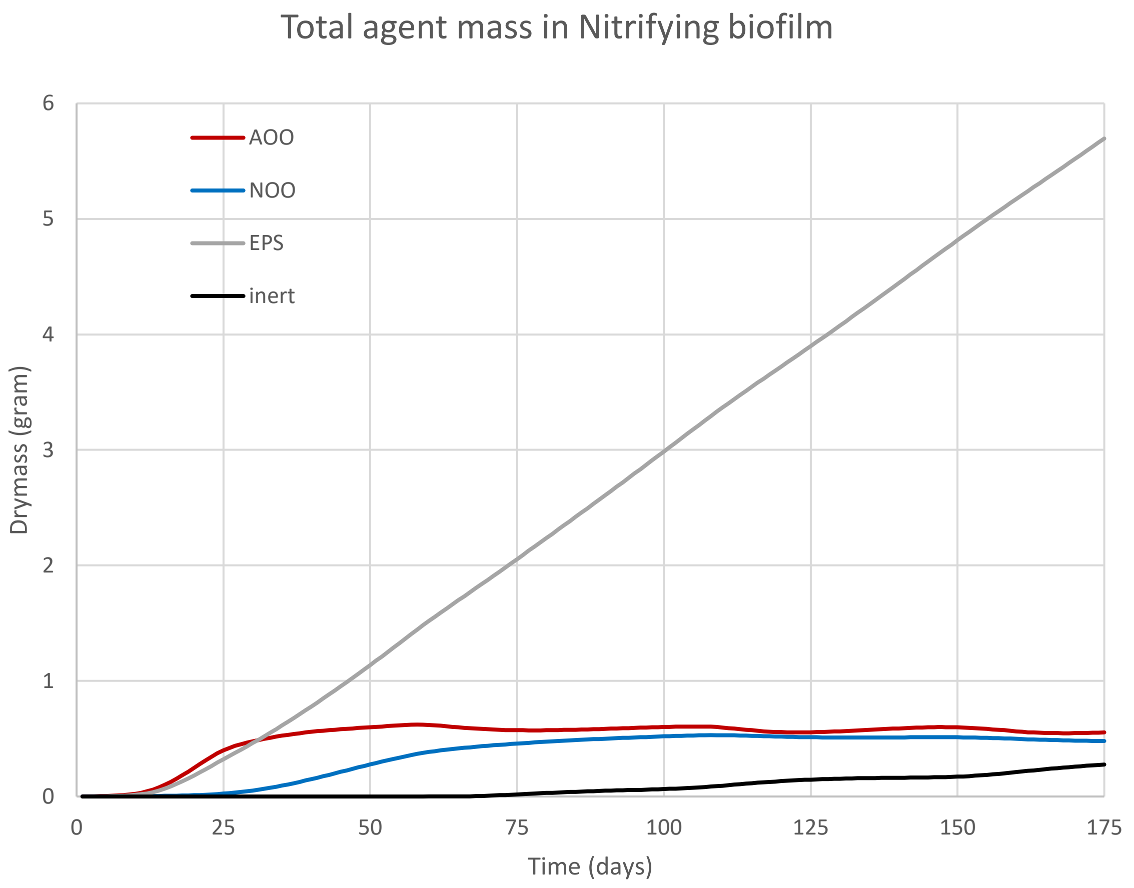

Fig C. Agent mass in the large-scale stress test simulation of a biofilm in 3D. The autotrophic nitrifying biofilm was initiated with 1 mg Ammonium Oxidizing Organisms (AOO, red) and 1 mg Nitrite Oxidizing Organisms (NOO, blue). Both species produce EPS particles (gray). Agents that drop below 20% of their division mass as a result of endogenous respiration/decay became inactive (black). At 175 days, the biofilm contains 1.02×10^7 agents.

Table E. Petersen (stoichiometric) matrix for reactions in the stress test.

	S_{NH4}	S_{O2}	S_{NO2}	S_{NO3}	X_{AOO}	X_{NOO}	EPS	Kinetic expression
	gN m^{-3}	*gCOD m*$^{-3}$	*gN m*$^{-3}$	*gN m*$^{-3}$	*gCOD m*$^{-3}$	*gCOD m*$^{-3}$	*gEPS m*$^{-3}$	
AOO growth	$-i_{NXB} - \frac{1}{Y_{AOO}}$	$-\frac{3.43 - Y_{AOO}}{Y_{AOO}}$	$\frac{1}{Y_{AOO}}$		1		$\frac{1}{3}$	$\mu_{AOO} * X_{AOO} * \frac{S_{NH_4}}{K_{NH_4,AOO} + S_{NH_4}} * \frac{S_{O_2}}{K_{O_2,AOO} + S_{O_2}}$
NOO growth	$-i_{NXB}$	$-\frac{1.14 - Y_{NOO}}{Y_{NOO}}$	$-\frac{1}{Y_{NOO}}$	$\frac{1}{Y_{NOO}}$		1	$\frac{1}{3}$	$\mu_{NOO} * X_{NOO} * \frac{S_{NO_2}}{K_{NO_2,NOO} + S_{NO_2}} * \frac{S_{O_2}}{K_{O_2,NOO} + S_{O_2}}$
AOO decay					-1			$b_{AOO} * X_{AOO}$
NOO decay						-1		$b_{NOO} * X_{NOO}$

S1.5 Benchmark 3, a comparison of biofilm modeling platforms

One of the longest established applications of biofilm modeling is modeling the treatment of wastewater. The International Water Association (IWA) set up a Biofilm Modeling Task group to compare computational modeling approaches to biofilms and provide guidance for researchers seeking to simulate biofilms. One of the key outputs of this work was the development of a series of Benchmark models – biofilm systems that could be modeled in a variety of modeling platforms to facilitate comparisons between different modeling approaches and establish the effects of different model designs and simplifying assumptions on simulation outputs [3]. The most complex of a set of benchmarks, Benchmark 3 (BM3), was designed to simulate microbial competition in a biofilm, with a source of chemical oxygen demand (COD) being oxidized by a population of heterotrophs and a population of autotrophs oxidizing ammonia to nitrate. This can be thought of as lumping two-step nitrification by ammonia- and nitrite-oxidizing organisms into a single process or modeling one-step nitrification by comammox (complete ammonia oxidizers). BM3 is necessarily limited to what all models are capable of simulating.

The IWA Biofilm Modeling Task Group ran BM3 simulations on a wide range of modeling platforms, with a variety of different approaches to modeling biofilms. Later, BM3 was also used for model validation in the development of iDynoMiCS [4] and NUFEB [5]. Here, iDynoMiCS 2.0 is compared against a selection of four models from the original IWA task group, as well as NUFEB and iDynoMiCS 1. A summary of the different models and their approaches to BM3 follows:

- W – a one-dimensional continuum biomass model run on the AQUASIM software [6] and developed by Peter Reichert and Oskar Wanner [7,8]
- M1 – a variant of the W model with a fixed boundary-layer thickness by Eberhard Morgenroth et al. [9]
- DN – a two-dimensional cellular automaton model developed by Daniel Noguera and colleagues [10]
- CP – a two-dimensional individual-based model, with biomass spreading via shoving, developed by Cristian Picioreanu and colleagues [11]
- NUFEB – A three-dimensional individual-based model that uses a platform derived from a molecular dynamics simulator by Li et al. [5]
- iDynoMiCS 1 – An individual-based model by Lardon et al. [4] used here for 2D simulations. This platform is the precursor to the one described in this paper, and the implementation of BM3 is very similar

As this set of modeling platforms represents a variety of different modeling approaches, they provide a valuable set of results against which to compare iDynoMiCS 2.0. The BM3 scenario has previously been used by Lardon et al. [4] and Li et al. [5] to benchmark iDynoMiCS 1 and NUFEB, respectively. Note that NUFEB has also been directly compared with iDynoMiCS 1 based on the BM3 scenario but varying seven model parameters sampled with a Latin hypercube.

As this set of modeling platforms represents a variety of different modeling approaches, they provide a valuable set of results against which to compare the results of the BM3 simulation in iDynoMiCS 2.0. A description of the implementation of the BM3 model in iDynoMiCS 2.0 follows, henceforth referred to by the abbreviation BM3-iD2.

BM3-iD2 Model Description

Previous descriptions of BM3 did not explicitly state two critical details that we had to infer by trial and error. One was that the oxygen concentration in the bulk liquid was kept constant and the other was

that the biomass density of the biofilm had to be tuned by scaling the biomass density of the agents. Hence, to facilitate reproduction, we give a full description of BM3 here, using the ODD protocol as a framework, with parts of the description that are already covered by the ODD description of iDynoMiCS 2.0 omitted. The description of BM3-iD2 follows the description of BM3 in Wanner et al. [3].

Overview

Purpose and patterns

This model simulates multi-species biofilms growing in an aqueous environment as commonly found both in nature and in treatment systems for wastewater and drinking water. The biofilm is composed of two species representing microbial functional groups – an aerobic heterotroph and an aerobic autotrophic nitrifier. Both of these species undergo inactivation processes which transform an agent's active biomass to inert biomass, meaning that there are three types of biomass present in the biofilm: heterotrophic, autotrophic and inert. The two microbial species compete for oxygen and for space in the biofilm and are transformed into the same inert biomass, leading to vertical stratification of the three different types of biomass through the biofilm.

The purpose of the BM3-iD2 model is to allow comparison between iDynoMiCS 2.0 and other biofilm models. Previous publications did not report time series and only some reported biomass distributions, which limits comparisons to various characteristics of the steady state, including solute concentrations in the bulk liquid, biomass concentrations and to some extent biomass distribution. A close match to other implementations of BM3 would demonstrate that differences in biomass spreading mechanisms between the models have little impact on overall transformation and growth rates in the biofilms and suggest that iDynoMiCS 2.0 is a reliable modeling platform. Deviations would suggest that differences between models, primarily different biomass spreading mechanisms, could affect predictions of overall biofilm performance.

Table F. Parameters used in the Benchmark 3 simulations.

Parameter	Value		
	Standard case	High ammonium	Low ammonium
Ammonium influent concentration	6 g m^{-3}	30 g m^{-3}	1.5 g m^{-3}
Dilution rate	0.0111 min^{-1}		
Volume of bulk liquid	4.0 nL		
COD influent concentration	30 g m^{-3}		
Carrier surface area	320 μm^2		
Biofilm thickness	500 µm		
Constant oxygen concentration in bulk liquid	10 g m^{-3}		
Biofilm density	10 g L^{-1}		
Agent density (Shoving)	12.5 g L^{-1}		
Agent density (FbM)	10.08 g L^{-1}		
Agent division dry mass	4 pg		
Boundary layer thickness	0 µm		
Shove Factor	1.05		

Entities, State Variables and Scales

The computational domain for BM3-iD2 is a 2-dimensional, spatially explicit compartment with a width of 320 µm. As detailed in Submodels, 2D simulations have a virtual third dimension with a thickness of 1 µm, meaning the effective surface area at the base of the biofilm is 320 µm^2. This domain represents a vertical slice of a biofilm that contains all simulated microbial agents. In order to maintain the defined biofilm thickness of 500 µm, all agents with a central point greater than 500 µm above the base are removed from the simulation at the beginning of each simulated time step. The biofilm compartment is coupled to a well-mixed bulk liquid compartment with a volume of 0.4 µL, which receives a constant inflow of 0.26 µL h^{-1}, with outflow of the bulk liquid at the same rate. Inflowing bulk liquid contains three solutes at fixed concentrations: organic carbon measured as chemical oxygen demand (COD) at 30 g m^{-3}, oxygen at 10 g m^{-3} and ammonium at three different concentrations (Table F). Solutes are well-mixed in the bulk compartment and in the upper portion of the spatial domain above the boundary layer. In the portion of the spatial domain that contains the boundary layer and biofilm, solutes diffuse through a grid with a resolution of 20 µm. The principal agents in BM3-iD2 are the microbial agents, of which there are two types – autotrophs and heterotrophs. Both species are modeled as spherical cells (coccoids), with a division mass of 4 pg. Agent biomass is composed of active and inert portions for both species.

Most models in the original IWA task group could directly set a biofilm biomass density as a parameter, and this is defined in BM3 as 10 g L^{-1}. However, as iDynoMiCS-2 is an individual-based model, users can only set the density of agents, with biofilm density an emergent property. In order to match the biofilm density in other models, simulations were run with a variety of agent densities until a biofilm density matching the other models was obtained. Since the emergent biofilm density depended on the agent relaxation method used, in simulations using shoving, an agent (cellular) biomass density of 12.5 g L^{-1} was used, while in simulations using Force-based Mechanics, an agent biomass density of 10.08 g L^{-1} was used. It was also discovered that the biofilm density used when running BM3 in the original iDynoMiCS 1 was incorrectly stated in the publication [4] as an agent biomass density of 15 g L^{-1}, but this led to a final biofilm density of ~ 12 g L^{-1}. Hence these simulations were rerun with the modified agent density used in BM3-iD2 to match a biofilm density of 10 g L^{-1}. These new results in iDynoMiCS 1 are also presented here.

Process Overview and Scheduling

The BM3-iD2 simulation proceeds in global timesteps representing 12 minutes of simulated time. Within this timestep, various core processes are simulated in a set order, while other processes (specifically, data reporting processes) occur less regularly than the global timestep. The order of processes in the spatial domain is as follows:

1. Agent removal – Agents with centers higher than 500 µm above the base of the biofilm are removed
2. Mechanical relaxation – Either shoving or Force-based Mechanical relaxation to minimize agent overlaps
3. Reaction-diffusion – Agents determine their reaction rates, based on solute concentrations and biomass amounts. Active agents also grow and divide. Solute concentration grids are updated according to reaction rates, and the boundary with the bulk compartment is updated (see *Submodels*)
4. Reporting (only every 2 simulated hours) – Biomass density grids and totals of different biomasses are written to files

In the bulk compartment, there is a simpler series of processes as follows:

1. Solute concentrations are updated according to inflows, outflows and diffusion into the biofilm (as determined by the boundary between the two compartments)
2. A file recording solute concentrations is updated.

These two sets of processes are carried out separately within each timestep, with the bulk compartment carrying out its processes before the biofilm compartment.

Design Concepts

The majority of the design concepts in BM3-iD2 are identical to the design concepts of the modeling platform itself, iDynoMiCS 2.0. Therefore, for a fuller description of the design concepts, see the Methods section of this paper. Design concepts that are specific to the BM3-iD2 model are described below.

Emergence: The interactions between the two species, especially the competition for oxygen and for space, because the top of the biofilm is maintained at a constant height, lead to particular distributions of biomass within the biofilm, which in turn determine the steady state concentrations of COD and ammonium in the bulk liquid.

Interaction: Agents interact with one another and with solutes in their local environment. Physical interactions cause agents to push against one another as they grow, causing a flow of actively growing agents and their neighbors upwards towards the top of the biofilm. Consumption of solutes by agents determines the rates of solute diffusion into the biofilm and also facilitates competition between agents, with agents near the top of the biofilm having access to solutes at greater concentrations.

iDynoMiCS 2.0 has two main agent overlap relaxation methods: A Shoving algorithm and Force-based Mechanics, described in detail in *Submodels*. In order to establish whether these different relaxation methods affected the results of BM3, simulations were run with both methods, with agent density adjusted for each method, to achieve an overall biofilm density of 10 g L^{-1}.

Table G. Petersen (stoichiometric) matrix for reactions in the Benchmark 3 simulations, adapted from Rittmann *et al.* [12] and Lardon *et al.* [4]. Biomasses are denoted with X. Specifically, X_H = heterotroph active biomass, X_N = nitrifier (autotroph) active biomass. Substrate concentrations are denoted with S, S_S for the organic substrate COD, S_N for ammonium and S_{O2} for oxygen. For descriptions of the other parameters, see Table H.

	Biomass type		Substrate			Kinetic expression
	Active	Inert	S_S	S_N	S_{O2}	
Heterotroph growth	1		$\frac{-1}{Y_H}$		$\frac{-(1-Y_H)}{Y_H}$	$\mu_{max,H}\frac{S_S}{K_S+S_S}\frac{S_{O2}}{K_{O2,H}+S_{O2}}X_H$
Heterotroph decay	-1	1				$b_{ina,H}X_H$
Heterotroph maintenance	-1				-1	$b_{res,H}X_H\frac{S_{O2}}{K_{O2,H}+\ S_{O2}}$
Autotroph growth	1			$\frac{-1}{Y_H}$	$\frac{-(4.57-Y_N)}{Y_N}$	$\mu_{max,N}\frac{S_N}{K_N+S_N}\frac{S_{O2}}{K_{O2,N}+S_{O2}}X_N$
Autotroph decay	-1	1				$b_{ina,N}X_N$
Autotroph maintenance	-1				-1	$b_{res,N}X_N\frac{S_{O2}}{K_{O2,N}+\ S_{O2}}$

Initialization

50 agents of each species are placed randomly within the bottom 160 µm of the spatial compartment before the first timestep of the simulation. Each of these agents starts with 10 pg active biomass, meaning they are expected to divide in the first timestep as the division mass is 4 pg, introducing some stochastic variation in total agent masses. Initial solute concentrations are set to the values in the bulk inflow.

Submodels

Bulk solute dynamics

The concentrations of COD and ammonium are solved in the bulk compartment according to Equation 3. However, the concentration of oxygen in the bulk had to be fixed at 10 g m^{-3} to match the results reported for BM3. In the well-mixed region of the spatial compartment that does not contain any agents, concentrations are set to those in the bulk compartment. In the rest of the spatial domain, solutes diffuse through the solute grid and are consumed by agents at rates according to the agent reactions.

Table H. Kinetic parameters in the Benchmark 3 models.

Parameter	Symbol	Value
Maximum specific growth rate, heterotroph	$\mu_{max,H}$	5.9976 day^{-1}
Half-saturation constant, heterotroph growth	K_S	4 g m^{-3}
Heterotroph growth yield	Y_H	0.63
Half-saturation constant, heterotroph maintenance	$K_{O2,H}$	0.2 g m^{-3}
Maintenance rate, heterotroph	$b_{res,H}$	0.32 day^{-1}
Decay rate, heterotroph	$b_{ina,H}$	0.08 day^{-1}
Maximum specific growth rate, autotroph	$u_{max,N}$	0.1386 day^{-1}
Half-saturation constant, autotroph growth	K_N	1.5 g m^{-3}
Autotroph growth yield	Y_N	0.063
Maintenance rate, autotroph	$b_{res,N}$	0.12 day^{-1}
Decay rate, autotroph	$b_{ina,N}$	0.03 day^{-1}
Half-saturation constant, autotroph maintenance	$K_{O2,N}$	0.5 g m^{-3}

Agent Reactions

Both species carry out three different reactions, a metabolism reaction, a maintenance reaction and an inactivation reaction. The metabolism reaction is equivalent to growth, increasing biomass, and using COD as electron donor and oxygen as electron acceptor in the case of heterotrophs and ammonium as electron donor and oxygen as electron acceptor in the case of autotrophs. The maintenance reaction represents the endogenous respiratory consumption of biomass for cell maintenance and oxidizes biomass with oxygen. The inactivation reaction lumps any additional loss of active biomass into a single decay process which converts metabolically active biomass to inert biomass. The growth and respiration reactions proceed according to Monod kinetics, while the inactivation reaction is governed by first order kinetics. See Tables G for the Petersen matrix and Table H for the kinetic parameters.

BM3-iD2 Results

Once parameters were finalized, three replicates of each combination of case and relaxation method were simulated in iDynoMiCS 2.0 for 120 simulated days. Additionally, three replicates of each case were simulated in iDynoMiCS 1 with the newly adjusted agent density parameter for the purposes of comparison. Solute concentrations generally reached steady state within 20 simulated days, while biomass took longer to reach steady state (Fig D). To compare iDynoMiCS 2.0 to the other models that have run BM3, various output variables were compared (Figs D, E, Table I). These included steady state concentrations of COD and ammonium, steady state densities of the various biomass forms and the distribution of biomass within the biofilm.

Solute Concentrations

Unsurprisingly, the BM3 results from iDynoMiCS 1 and iDynoMiCS 2.0 were very similar (Fig 4). These models have very similar basic designs and in a simple biofilm model, behave very similarly. Furthermore, there is no clear impact of the biomass spreading mechanism used in iDynoMiCS 2.0 as the Shoving or Force-based Mechanics simulation results were very similar. However, different agent densities were required for these two spreading methods to produce an overall biofilm density of 10 g L^{-1}, because the FbM produced denser biofilms than the Shoving algorithm in the absence of this adjustment. This is because Shoving is generally used to model the effect of EPS production – increased distance between cells – implicitly by using a Shoving factor, a multiplier on the radius of the cells. There is no EPS in BM3, including EPS would reduce biofilm density in a mechanistic way.

Steady state bulk liquid concentrations were compared with the results from the other models using the multivariate version of the t-test, Hotelling's 1-sample T^2 test. Results from iDynoMiCS 2.0 did not differ significantly from the distribution of results of the other models, whether using the Shoving relaxation algorithm or the Force-based Mechanics (Table I). Despite this, the steady state COD concentrations in iDynoMiCS 1 and 2.0 were generally higher than those in most of the other models (Fig 4, Table I).

Biomass distribution

Another key output of the BM3-iD2 model is the biomass density and vertical distribution. As both species in the model can have both active and inert biomass, there are three different biomass types with different concentrations and distributions - heterotrophic, autotrophic and inert. The total areal densities of these biomass types are compared between models in Table J. Vertical distributions of the various biomass types in the different cases are shown in Fig F. These show a qualitatively similar pattern to the CP model [13], with fast-growing heterotrophs dominating the top of the biofilm, while autotrophs grow more slowly and are at their most abundant in the middle or bottom of the biofilm. Autotrophs vary widely in abundance between the different cases, being at very low numbers in the low ammonium case. This is to be expected, given that their energy source is at a low concentration. In all three cases, the bottom of the biofilm is dominated by inert biomass due to the lower substrate concentrations at the bottom of the biofilm reducing growth relative to maintenance and inactivation. This is most pronounced in the low ammonium case, which has the highest proportion of inert biomass of the three cases.

Given the differences in modeling approaches of the various IWA task group models, one might expect iDynoMiCS 1 and iDynoMiCS 2.0 to produce results closer to the NUFEB and CP models than to any of the other IWA task group models. Although the NUFEB results were close, differences with CP are larger. In fact, the results from the W platform were the closest match in steady state solute concentrations, while those from the M1 platform were the closest match for overall biomass densities. This is particularly interesting given that these are both 1-dimensional platforms utilizing the AQUASIM software rather than agent-based. It is possible that the similarity derives from a closer

match in biomass distribution than with the other models due to less stochastic mixing of the biomass. However, as the biomass distributions were not published for these models, this is difficult to determine. Hotelling's 1-sample T^2 tests were carried out to compare the areal biomass densities in iDynoMiCS 2.0 to that in the IWA models. The results from iDynoMiCS 2.0 do not differ significantly from the set of results from the IWA models.

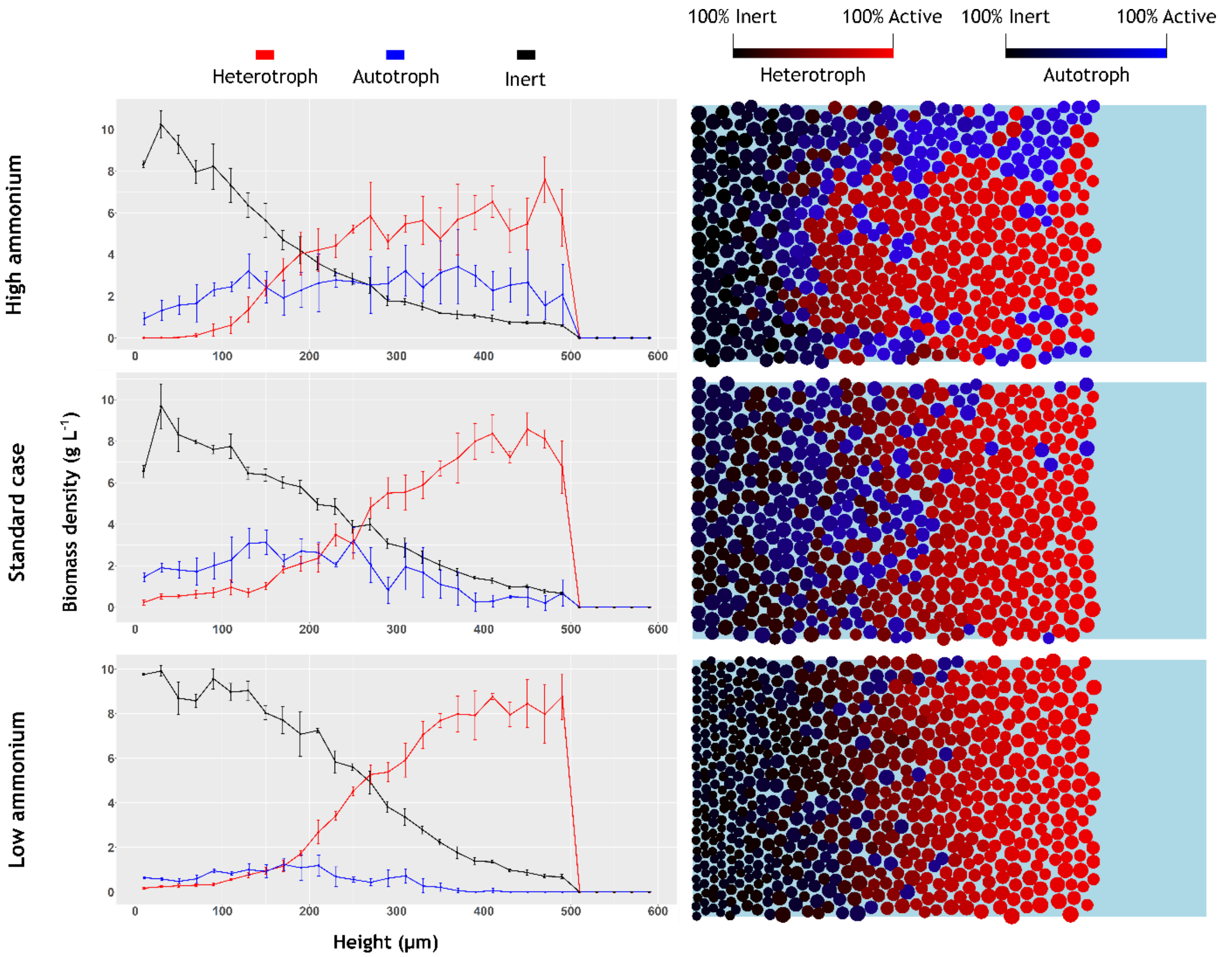

Fig D. BM3-iD2 Spatial biomass distribution in the three cases using shoving. Left column shows the average areal density of each biomass type. Error bars show the standard deviation based on the three replicates run for each case. Right column shows an example from the final timestep of one replicate for each case. Results are from the simulations with the shoving biomass spreading algorithm. Coloring of agents shows the proportion of biomass that is active (bright) or inert (dark). Time courses are shown in Fig E.

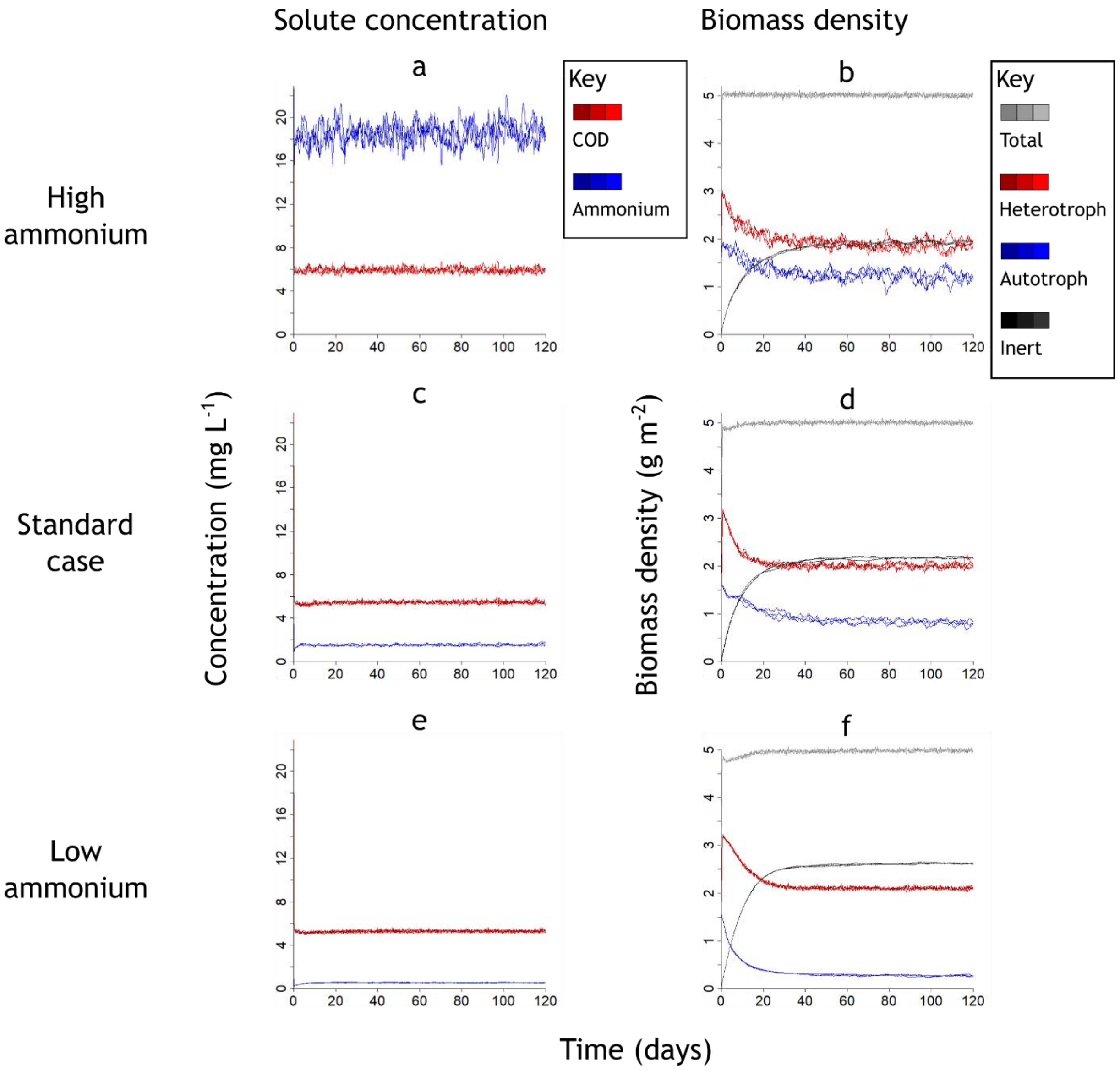

Fig E. BM3-iD2 solute concentrations and areal biomass densities over time in the three simulated cases. Lines of different shades of the same color represent different replicates of the simulations run with different random number seeds. Three replicates were run for each case. Results are from the simulations with the shoving biomass spreading algorithm. Spatial biomass distributions are shown in Fig D.

Table I. Steady state substrate concentrations in the various IWA task group models and in iDynoMiCS 1 and iDynoMiCS 2.0. Results for the latter models were averaged over the stochastic steady states. Hotelling's T^2 tests were performed to compare the results from iDynoMiCS 2.0 to those from all other models, including the IWA models, NUFEB and iDynoMiCS 1.

		High ammonium		Standard case		Low ammonium	
		COD (mg L^{-1})	Ammonium (mg L^{-1})	COD (mg L^{-1})	Ammonium (mg L^{-1})	COD (mg L^{-1})	Ammonium (mg L^{-1})
IWA models	CP	5.45	18.15	5.14	1.50	4.39	0.44
	DN	5.56	20.26	5.14	1.74	4.98	0.48
	W	5.86	18.93	5.39	1.59	5.19	0.48
	M1	5.35	17.03	4.84	1.45	4.66	0.45
NUFEB		5.74	18.42	5.21	1.72	5.18	0.53
iDynoMiCS models	iDynoMiCS 1	6.08	18.58	5.63	1.55	5.45	0.55
	**iDynoMiCS 2.0 (Shoving)**	5.92	18.63	5.46	1.55	5.28	0.54
	Hotelling's T^2 Test p-value	0.2343		0.1743		0.0831	
	**iDynoMiCS 2.0 (FbM)**	5.89	18.05	5.41	1.49	5.20	0.53
	Hotelling's T^2 Test p-value	0.1575		0.1247		0.2173	

Table J. Steady state areal biomass density (mass per unit surface area) of different types of biomass in the biofilm. Hotelling's T^2 tests were performed to compare the results from iDynoMiCS 2.0 to those from the IWA models. Biomass density was not reported in the NUFEB model benchmark, and it is thus not included in this comparison.

		**Heterotroph density (g m^{-2})**	**Autotroph density (g m^{-2})**	**Inert density (g m^{-2})**
**High ammonium**	CP	1.71	1.07	2.42
	DN	2.92	1.10	0.98
	W	1.73	1.07	2.20
	M1	1.83	1.24	1.93
	**iDynoMiCS 2.0 (Shoving)**	**1.87**	**1.21**	**1.92**
		**Hotelling's T^2 test p-value**	**0.724**	
	**iDynoMiCS 2.0 (FbM)**	**1.77**	**1.29**	**1.92**
		**Hotelling's T^2 test p-value**	**0.4757**	
**Standard case**	CP	1.81	0.72	2.60
	DN	2.88	0.68	1.44
	W	1.88	0.79	2.33
	M1	2.02	0.83	2.15
	**iDynoMiCS 2.0 (Shoving)**	**2.01**	**0.82**	**2.17**
		**Hotelling's T^2 test p-value**	**0.7513**	
	**iDynoMiCS 2.0 (FbM)**	**2.00**	**0.83**	**2.13**
		**Hotelling's T^2 test p-value**	**0.6337**	
**Low ammonium**	CP	2.11	0.23	2.73
	DN	2.96	0.13	1.91
	W	2.00	0.21	2.80
	M1	2.14	0.21	2.65
	**iDynoMiCS 2.0 (Shoving)**	**2.10**	**0.27**	**2.62**
		**Hotelling's T^2 test p-value**	**0.1056**	
	**iDynoMiCS 2.0 (FbM)**	**2.10**	**0.27**	**2.60**
		**Hotelling's T^2 test p-value**	**0.09726**	

S1.6 Supplementary Information for "Comparing the effect of different biomass spreading mechanisms: Biofilms promote altruism case study"

Table K. Model parameters for 3D simulations of the "biofilms promote altruism" case study

**Description**	**Symbol**	**Value**	**Unit**	**Reference/notes**
Global timestep	Δt	1	h	Kreft 2004
Total simulation	t_{max}	21	d	Kreft 2004
Coccoid				
Agent density	ρ_x	0.1363	pg fL^{-1}	Adjusted to match biofilm density (original 0.29 pg fL^{-1})
Division threshold	x_{max}	0.08	pg	Chosen, 0.08 pg as this results in approximately the same cell volume used in BacSim
Filament				
Agent density	ρ_x	0.1363	pg fL^{-1}	Chosen
Division threshold	x_{max}	0.14	pg	Chosen
Transition threshold	$x_{transition}$	0.09	pg	Chosen
Spine stiffness	k_{spine}	0.56	fN	Chosen
Rod radius	r_{rod}	0.37	µm	Chosen
Connecting spring stiffness	$k_{connect}$	0.2778	fN	Chosen
Torsion spring stiffness	$k_{torsion}$	0.2778	fN	Chosen
Detachment probability	P(detach)	0.1		Chosen
**Yield strategist**				
Kox	K_{ox}	0.3	mg L^{-1}	Kreft 2004
Vmax	V_{max}	0.55836	h^{-1}	
Biomass per reaction	Y_x	0.147	gX gN^{-1}	Kreft 2004
Oxygen per reaction	Y_{ox}	-3.19565	gOx gN^{-1}	Kreft 2004
**Rate strategist**				
Kox	K_{ox}	0.6	mg L^{-1}	Kreft 2004
Vmax	V_{max}	2.23344	h^{-1}	
Biomass per reaction	Y_x	0.0735	gX gN^{-1}	Kreft 2004
Oxygen per reaction	Y_{ox}	-3.19565	gOx gN^{-1}	Kreft 2004
Domain				
Length x	l_x	200	µm	Kreft 2004
Length y	l_y	200	µm	Kreft 2004
Length z	l_z	12.5	µm	Chosen (original 2 µm)
Distance between solute nodes	res	25/16	µm	Chosen (original 2 µm)
Diffusion boundary layer	$l_{diffusion}$	40	µm	Kreft 2004
**Solutes**				
Initial oxygen concentration	S_{ox_init}	1	mg L^{-1}	Kreft 2004
Oxygen diffusivity	D_{ox}	2.0×10^{-5}	cm^2 s^{-1}	Kreft 2004
**Chemostat**				
Volume	$V_{chemostat}$	1	mm^3	Chosen
Flowrate	$Q_{chemostat}$	0.06	mm^3 h^{-1}	Chosen

Shoving as used in BacSim results in more open space between agents compared to mechanical relaxation as used in iDynoMiCS 2.0, which by default only resolves overlap between agents. To compensate for this effect, we have reduced agent density in iDynoMiCS 2.0 by 53%, such that overall biofilm densities remain similar. In order to compare biofilm densities, the computational domain was split into 100x100 bins. Each bin receives a number equal to the total mass of all agents with their center of gravity in the bin, division by the bin volume reveals the local biofilm density (Fig F).

For both the iDynoMiCS 2.0 and BacSim simulations, inspection of biofilm density at different heights revealed that the majority of bin rows were not significantly different in terms of density in comparison to the total amount of filled bins. For both platforms we observed a significant drop-off of biofilm density at the top surface where bins may be partially filled and the expansion front may result in a sparser local agent population.

In BacSim simulations we observe a significant biofilm density drop in the first row of bins (at the base of the biofilm). This may be a result of how the BacSim shoving algorithm resolves interactions with a hard surface in combination with less efficient sphere packing at a flat surface. No significant biofilm density drop was observed at the base of the iDynoMiCS 2.0 simulations. With both platforms, occasional small but significant peaks or drops in biofilm density (2.5 < P < 5.0) are observed in some bins. These occasional drops and peaks are likely artifacts as a result of a spatial aliasing effect between the binning resolution and local sphere packing.

With both platforms, density bins, excluding bins at the biofilm extremes, follow a normal distribution. After the before-mentioned agent density adjustment, no significant difference in the overall biofilm density is observed between simulations of the two platforms. The standard deviation of density bins is higher in BacSim simulations. This can be explained by the difference in agent properties, maximum agent size is kept the same in iDynoMiCS 2.0, translating into a higher overall amount of agents with a lower mass, resulting in less bin-to-bin mass fluctuation.

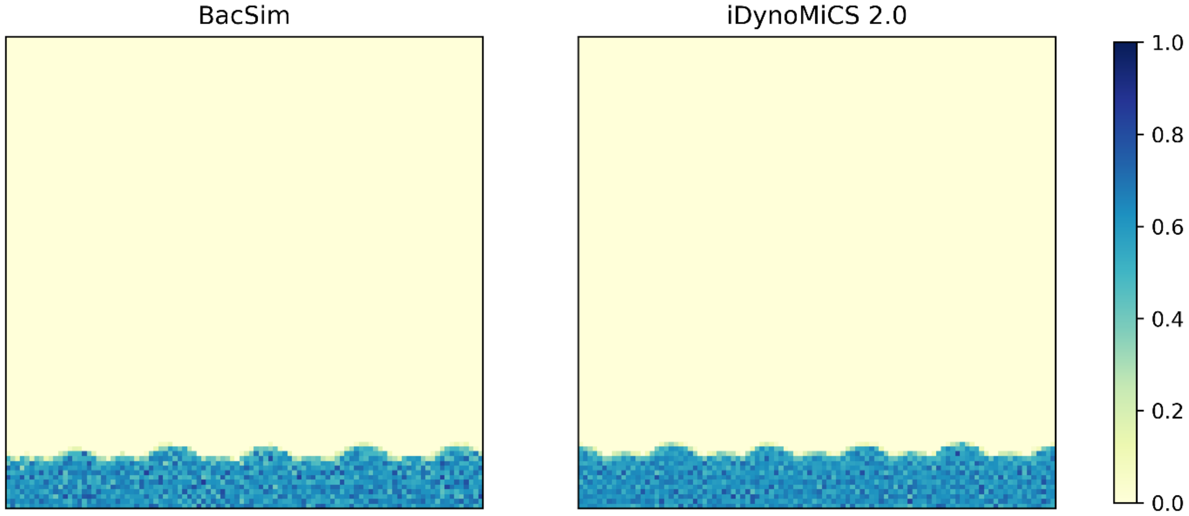

Fig F. Comparison of agent density (pg µm^{-3}) distributions in biofilms simulated in BacSim using the shoving algorithm vs iDynoMiCS 2.0 using FbM. The panels correspond to Fig 5a (left) and 5b (right). The computational domain was split into 100x100 grid elements, each received the full mass of agents whose center of gravity was inside the grid element, division by the grid element volume gave the local biofilm density (pg µm^{-3}). iDynoMiCS 2.0 agent density was reduced by 53% in order to achieve a similar overall biofilm density. With BacSim the biofilm density at the base was observed to be significantly lower than in the rest of the biofilm. The BacSim simulations further showed a higher standard deviation amongst bins, due to the higher agent density in these simulations.

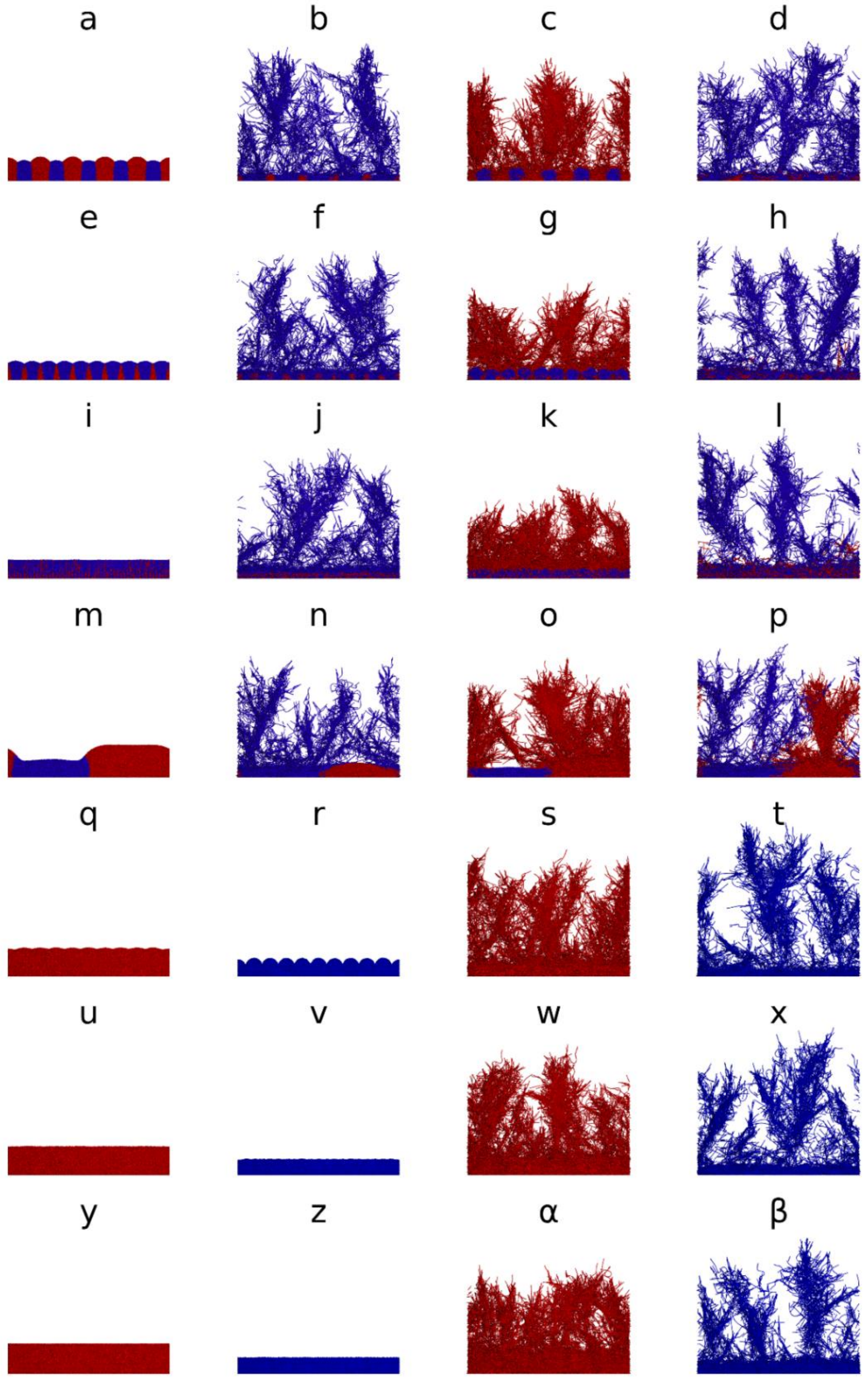

Fig G. Replicates of biofilms promote altruism case study simulations shown in Fig. 6. Rate Strategists (RS, blue) and Yield Strategists (YS, red) competed in a 3D biofilm domain (200x200x12.5 μm) for 3 weeks. In the first 4 rows, different strategies competed. Column 1 corresponds to spherical cell scenarios in Fig 2 of Ref [14] but were now simulated in 3D. In column 2, RS formed filaments and in column 3, YS formed filaments. Filaments won regardless of strategy. In column 4, both formed filaments and RS won or likely won. The last 3 rows show single species 'controls' with 10, 20 or 100 initial agents. The first two columns show simulations with spherical YS or RS agents while the last two columns show filament forming YS or RS agents. The filamentous microbes incorporate a basic life cycle in which initially spherical agents extend into rod shaped agents to further extend into a multi-segmented filaments as described in S1.14.

S1.7 Model initiation

Box A. Example of a simple iDynoMiCS 2.0 protocol file used to specify a particular model to be read and executed by the platform.


```xml
<?xml version="1.0" encoding="UTF-8"?>
<document>
 <simulation name="simple_biofilm" outputfolder="../results" log="NORMAL">
  <timer stepSize="3 [h]" endOfSimulation="10 [d]" />
  <speciesLib>
   <species name="bacterium">
    <speciesModule name="coccoid" />
    <aspect name="reactions" class="InstantiableList">
     <list nodeLabel="reaction" entryClass="RegularReaction">
      <reaction name="growth">
       <expression value="mass*mumax*(carbon/(carbon+Ks))*((oxygen/(oxygen+Kox))">
        <constant name="Ks" value="2.4[g/m+3]" />
        <constant name="Kox" value="0.6[g/m+3]" />
        <constant name="mumax" value="2.05[d-1]" />
       </expression>
       <stoichiometric component="mass" coefficient="1.0" />
       <stoichiometric component="oxygen" coefficient="-18.0" />
       <stoichiometric component="carbon" coefficient="-4.2" />
      </reaction>
     </list>
    </aspect>
   </species>
   <species name="coccoid">
    <aspect name="density" class="Double" value="0.15" />
    <aspect name="surfaces" class="AgentSurfaces" />
    <aspect name="morphology" class="String" value="coccoid" />
    <aspect name="volume" class="SimpleVolumeState" />
    <aspect name="radius" class="CylinderRadius" />
    <aspect name="divide" class="CoccoidDivision" />
    <aspect name="divisionMass" class="Double" value="0.2 [pg]" />
    <aspect name="updateBody" class="UpdateBody" />
   </species>
  </speciesLib>
  <compartment name="biofilm-compartment">
   <shape class="Rectangle" resolutionCalculator="MgFASResolution" nodeSystem="true">
    <dimension name="X" isCyclic="true" targetResolution="2.0" max="32.0"/>
    <dimension name="Y" isCyclic="false" targetResolution="2.0" max="64.0">
     <boundary extreme="1" class="FixedBoundary" layerThickness="32.0">
      <solute name="carbon" concentration="1.0 [mg/l]" />
      <solute name="oxygen" concentration="8.74 [mg/l]" />
     </boundary>
    </dimension>
   </shape>
   <solutes>
    <solute name="carbon" concentration="1.0 [mg/l]" defaultDiffusivity="2000.0 [um+2/s]" biofilmDiffusivity="1500.0 [um+2/s]" />
    <solute name="oxygen" concentration="8.74 [mg/l]" defaultDiffusivity="2000.0 [um+2/s]" biofilmDiffusivity="1500.0 [um+2/s]" />
   </solutes>
   <spawn class="randomSpawner" domain="32.0, 1.0" priority="0" number="30" morphology="COCCOID">
    <templateAgent>
     <aspect name="species" class="String" value="bacterium" />
     <aspect name="mass" class="Double" value="0.2" />
    </templateAgent>
   </spawn>
   <processManagers>
    <process name="agentRelax" class="AgentRelaxation" priority="0" />
    <process name="PDEWrapper" class="PDEWrapper" priority="1" />
   </processManagers>
  </compartment>
 </simulation>
</document>
```

S1.8 Software structure

Box B. This example shows how with a few lines of code a new aspect class can be created. In this case it is a class that calculates a coccoid radius from its volume. Because here the abstract super class "Calculated" is extended, the newly written class integrates seamlessly in the framework as initialization and data handling is handled automatically.

```
Import …
/** Example of a basic calculated aspect that returns the radius of a coccoid agent in 3D */
public class SimpleCoccoidRadius extends Calculated {
 public Object get( AspectInterface agent ) {
  return ExtraMath.cubeRoot( agent.getDouble( AspectRef.agentVolume ) * 0.75 / Math.PI );
 } }
```

S1.9 Included test scenarios

Table L. A selection of test protocols that are included with iDynoMiCS 2.0.

Title	File	Description
Simple	simple.xml	Minimalistic protocol file testing basic functionality with default parameters.
Chemostat	chemostat.xml	A basic chemostat setup with a reaction occurring in the environment (non-agent mediated).
Fed-batch	fedbatch.xml	Basic fedbatch scenario, agents grow in a non-spatial compartment with constant "feed" increasing volume and supplying solutes.
Sensing	simple_ sensing.xml	Basic scenario with local solute sensing, agents "differentiate" when the signal surpasses a threshold concentration.
Conditional coloring	conditional_ colouring.xml	A basic nitrifying biofilm with conditional coloring, agents receive a color gradient based on the amount of internally stored eps.
Bacilli	bacilli.xml	A basic setup with 2 colonies of rod shaped agents merging.
Plasmid spatial	plasmid.xml	A basic setup testing plasmid transfer in a spatial compartment.
Plasmid chemostat	plasmid_ chemostat.xml	A basic setup testing plasmid transfer in a well-mixed environment.
Stress test	stress_test_ 7c.xml	A large scale nitrifying biofilm used to test the limits of iDynoMiCS 2.0.
Benchmark 3	/BM3/ (6 files)	A set of model scenarios corresponding the 3 different ammonium concentrations as analog to the benchmark 3 cases using FbM or shoving.
Altruism 2.5D	c10_ld_fb.xml, d10_ld_fb.xml, e10_ld_fb.xml	A set of model scenarios corresponding to those found in "Biofilms promote altruism" Kreft 2004, Fig 4. scenario c, d and e.
Altruism 3D	/altruism 3D/ (28 files)	A set of model scenarios corresponding to scenarios presented in Fig 6.
Reaction diffusion tests	/reaction diffusion/ (4 files)	A set of model scenarios corresponding to the reaction and diffusion tests as presented in S1.3
Unit tests	/unit-tests/ (7 files)	A set of protocols used for automated software and solver testing.

S1.10 The graphical user interface

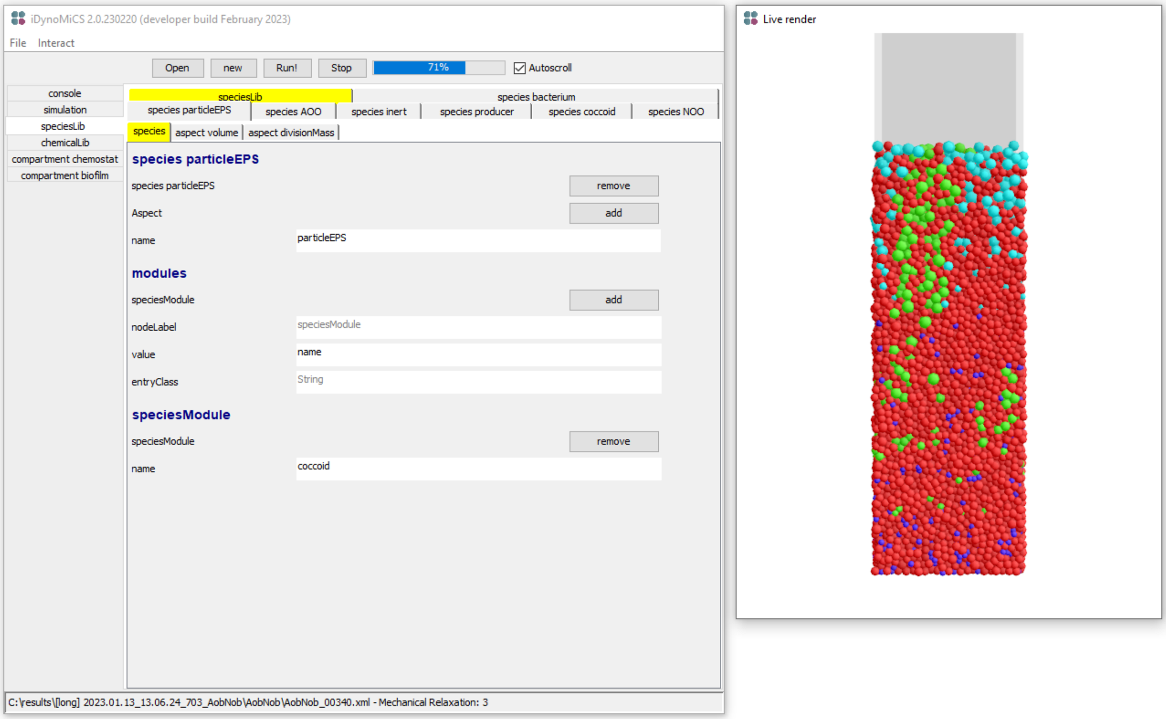

Fig H. Preview of the iDynoMiCS 2.0 GUI during simulation. The GUI may be used to review, edit or create protocol files before running them. During the simulation, the simulation state may be viewed but no longer edited (left). The GUI can further provide useful feedback including key information such as substrate concentration, species abundance, convergence of the reaction diffusion solver, etc. through the console. Spatial compartments can be rendered directly to visualize agent distribution and concentration gradients (right). Lastly the GUI can be used to extract key data from iDynoMiCS 2.0 output files, convert between EXI and XML files and convert numbers between different unit systems including SI and iDynoMiCS 2.0 base units.

S1.11 Plasmid Dynamics

For plasmid dynamics, two distinct processes were implemented: conjugative transfer from donor to recipient cells and loss of the plasmid due to segregation. Plasmid loss can only happen upon cell division and hence was encoded as a probability. Conjugation was considered pili-driven, with a maximum length pili can reach before they start retracting. The dynamics of transfer were incorporated based on the live cell imaging by Clarke *et al.* (2008) [15].

From the imaging, certain aspects of F-pilus extension and retraction can be observed: During extension, the filament elongates from the base. A fully extended 4-μm pilus retracts completely. F-pili on the same cell are independently regulated, with about three pili growing and retracting asynchronously. The average time required for extension and retraction of the pili informed the extension and retraction speeds of the pili used in the plasmid dynamics process manager.

In the model, the conjugation process begins with pili extension, assuming pili extend in all directions from the cell surface. On encountering a recipient, the pilus tries to attach to its surface and if a pilus attaches to a recipient cell, all pili start retracting. Certain pili are capable of transferring the plasmid without pili retraction, but others transfer the plasmid only upon cell surface to surface contact, with the pilus only bringing the cells together by retracting with the recipient cell attached.

Like in iDynoMiCS 1, plasmid carrying cells search the neighborhood within the reach of the pilus. A difference arises in the method implemented for this search. Instead of the "scan speed" in iDynoMiCS 1, the required parameters are maximum pilus length and "transfer probability". Using the F-pili data from live cell imaging by Clarke *et al.* (2008), the pilus length can be calculated for each time step of the process as a function of extension speed. The current length is used as the maximum distance for neighborhood search. The closest neighbors are prioritized by increasing the neighbor search range in increments of 0.01 μm until the current pilus length is reached or a neighbor is found. As an example, with pilus length of 3.2 μm, a neighborhood search will be performed 320 times with the distance searched increased from 0.01 to 3.2 in increments of 0.01 μm. The search will be terminated early if a plasmid-free neighbor is found.

Once a plasmid free neighbor is found, the transfer event happens instantly with a success probability given by the parameter "transfer probability". Biologically, the transfer happens after pilus retraction and then the plasmid goes into a "cool down" period. To reduce the computational requirement for agent movements, the time for retraction of the longest pilus is added to the cool down period as a wait time between plasmid transfer attempts.

Table M. Parameters required for the plasmid dynamics process manager in iDynoMiCS 2.0.

<table>
<tr><th>Parameter</th><th>Definition</th></tr>
<tr><td>Transfer probability</td><td rowspan="2">Governs the success of plasmid transfer; user must provide either one of the parameters with the relation being:
Transfer probability = frequency × number of neighbors</td></tr>
<tr><td>Transfer frequency</td></tr>
<tr><td>Loss probability</td><td>Governs the success of loss event upon cell division</td></tr>
<tr><td>Pilus length</td><td>Maximum length of pilus extension from donor cell surface. If plasmid transfer is not pilus driven, this can be set to 0</td></tr>
<tr><td>Aspects to Transfer</td><td>Agent aspects to change on plasmid acquisition or loss (MIC, Fitness cost, etc.)</td></tr>
<tr><td>Cool down</td><td>Rest time between plasmid transfers</td></tr>
<tr><td>Extension speed</td><td>Extension speed for pilus related to this plasmid</td></tr>
<tr><td>Retraction speed</td><td>Retraction speed for pilus related to this plasmid. The time for complete retraction is added to the cool down time before the next transfer can start</td></tr>
</table>

Since each plasmid is a separate aspect specified in the protocol file, there can be multiple plasmids included. These will implicitly be considered compatible with each other as plasmid incompatibility is not currently implemented.

Plasmid loss due to segregation is defined as an event in the code, thus requiring inclusion as an aspect in the protocol file. However, the event is triggered upon cell division only if the "loss probability" parameter is defined in the included plasmid dynamics process manager. Thus, upon cell division, the daughter cells can retain the plasmid, or one can lose it at the probability defined in the protocol file.

For chemostats, the transfer process is governed by the following equation to determine the number of transfers for each agent with plasmid (donor):

$$\frac{\beta\, R}{R+1}\Delta t \tag{S14}$$

Where β is the transfer frequency, R is the number of plasmid-free agents (recipients) in the population and Δt is the time step size. This equation is calculated for each donor so implicitly, the rate is proportional to donor concentration. It is analogous to the infection rate in Susceptible-Infectious-Recovered compartmental epidemiological models, where recipients are considered Susceptible and plasmid transfer is analogous to the process of infection. The recipients for the plasmid are selected randomly from the whole population as a chemostat is considered well-mixed.

S1.12 Agent density scaling for 2D simulations

Due to the virtual third dimension of 1 µm in 2D simulations, cell radii and/or lengths can differ between 2D and 3D compartments. iDynoMiCS 2.0 can scale agent densities, such that the dimensions of agents in 2D match what they would be in a 3D environment.

Users define an actual 3D density, ρ_{3D}, which is then used to calculate a scaled density in the 2D compartment, ρ_{2D}. The exact calculation depends on the shape of the agent or filament section in question.

For spherical (coccoid) agents, the radius of a sphere is calculated based on the agent's mass and actual density:

$$r_c = \sqrt[3]{\frac{3}{4\pi}\frac{m}{\rho_{3D}}} \qquad \text{(S15)}$$

Where r_c is the radius and m the agent's total mass. The scaled density is thus given by:

$$\rho_{2D} = \frac{m}{\pi {r_c}^2} \qquad \text{(S16)}$$

For agents or filament elements with a rod (bacillus) shape, it is the length, rather than the radius, that must be calculated. The 3D length of the line-segment connecting the agent's points is given by:

$$l = (\frac{m}{\rho_{3D}} - \frac{4}{3}\pi {r_c}^3)/\pi {r_c}^2 \qquad \text{(S17)}$$

And the 2D scaled density is given by:

$$\rho_{2D} = m/(\pi {r_c}^2 + 2r_c l) \qquad \text{(S18)}$$

S1.13 Microbial IbM publications on PubMed

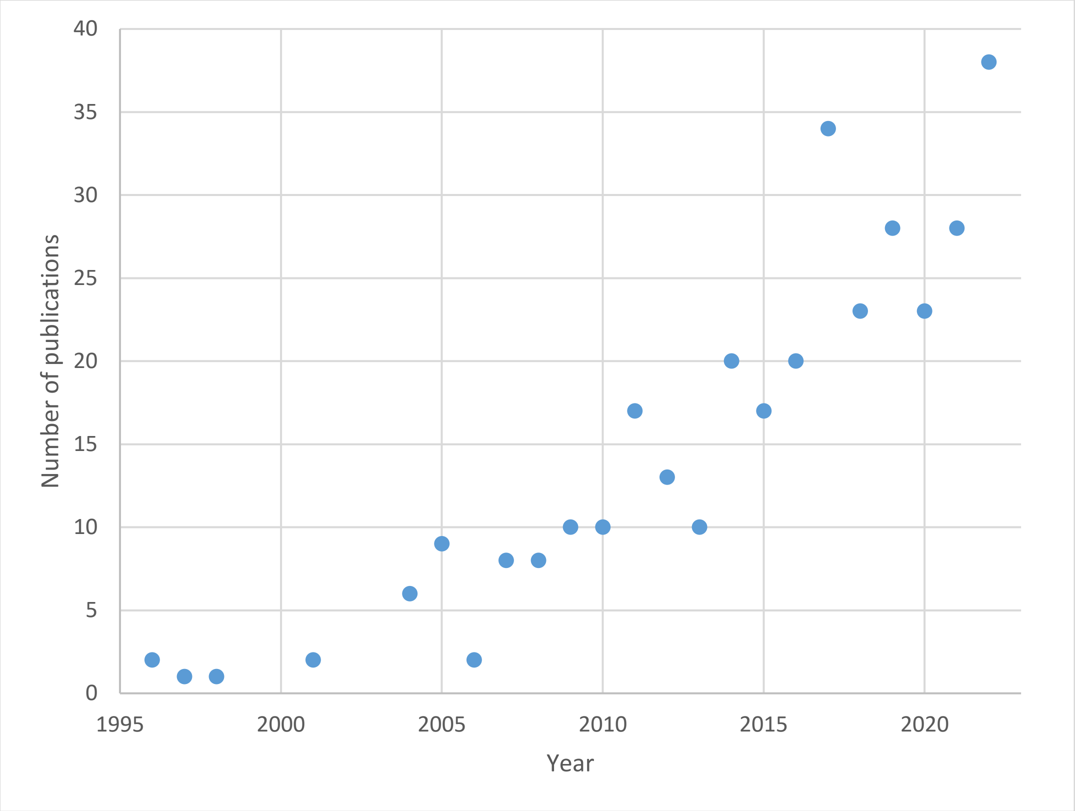

Fig I. The number of publications on microbial IbM on PubMed since 1995. A simple search query on PubMed reveals a growing trend in applying IbM to microorganisms. The following query was used: "((biofilm) OR (microbial)) AND ((individual-based) OR (agent-based)) AND (model)".

S 1.14 Division events

When an agent reaches the user set division mass threshold, a new agent is created and the mass of the original agent is distributed. By default, a small random deviation is incorporated where one cell receives 50% of the original mass plus a normally distributed value with a coefficient of variation of 5% (this can be changed in the protocol file).

When a single spherical agent divides, this will result in two agents with approximately half the mass of the original agent. The two resulting cells are placed with their centers of gravity half an agent radius (of the original agent) away from the original center of gravity in opposite directions, utilizing a random vector to determine this direction (Fig J).

When the agents form filaments, any subsequent divisions will take the positions of attached sibling cells into account for determining the direction of division. The new agents resulting from the division will be placed in between the original center of gravity and the center of gravity of the closest attached sibling in the filament, by default this position is set to be 5% of the original agent's radius away from the original agent's center of gravity (this value can be changed).

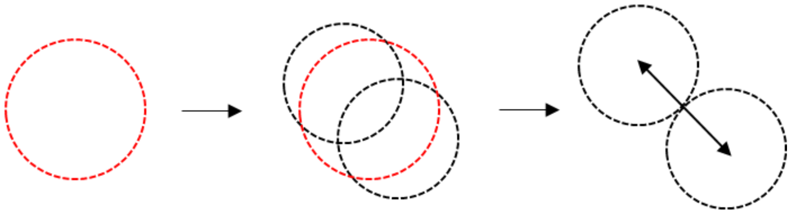

Fig J. Spherical agent division. Resulting agents are placed in opposite directions, residual overlap is resolved with FbM or shoving.

In the case of rod-shaped agents, the new agents are positioned by retaining the mass point positions of the original agent and placing two new mass points in between (one for each resulting agent). These new mass points are placed on opposite sides of the original agent's center of gravity, and further randomized using a random vector following a uniform distribution scaled to 5% of the agent's radius. This randomization can be configured in the protocol file. The process is the same for filament forming rods (Fig K).

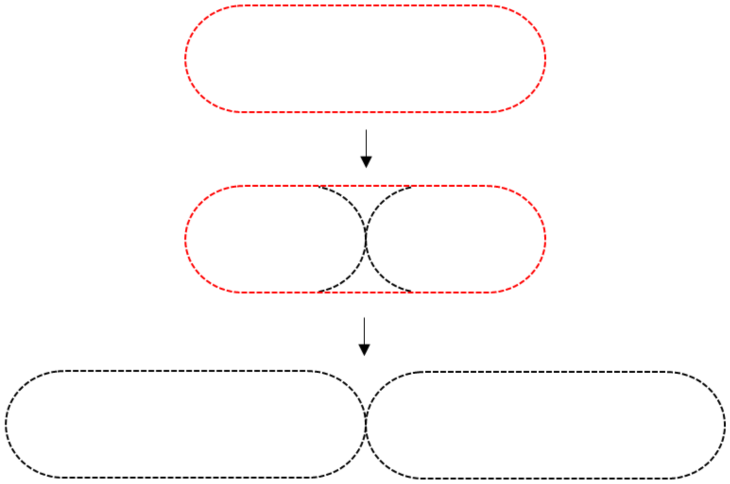

Fig K. Agent division with rod morphology. The two resulting agents initially take the same place as the original agent with their new mass points placed in between on opposite sides of the original agent's center of gravity. As a result, the new agents will be compressed, which is resolved by the FbM algorithm.

Finally, it is also possible to incorporate morphological shifts. In this scenario, small agents start with a spherical morphology. When the agent reaches a transition mass threshold, a rod containing two mass points is created (morphological shift 1). The placement of these mass points follows the same steps as with regular spherical agent division. When the rod grows to reach the division mass, two spherical agents are created at the same position as the rod's mass points (morphological shift 2). These agents can remain attached to form a filament, as the attached spherical agents reach their transition mass, their morphology shifts to a rod shape again with the positioning of the additional mass points following the same steps as the division of spherical cells in a filament.

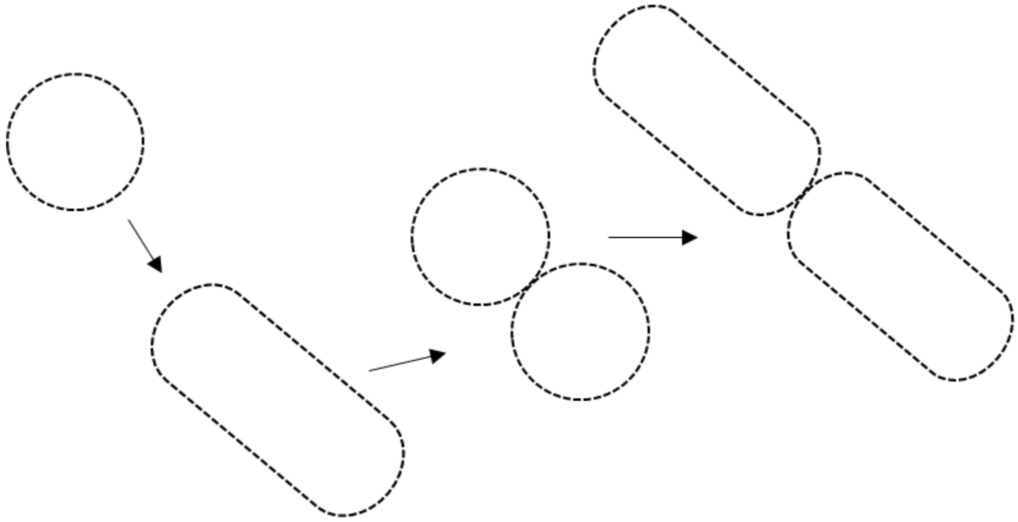

Fig L. An agent that incorporates morphological shifts in its growth process. The agent shape cycles from spherical to rod shaped and back to form two spherical agents upon division.

In all these cases, the placement of new agents will result in mechanical stresses due to agent overlaps. These mechanical stresses are resolved using mechanical relaxation following FbM.

S 1.15 SI References

1. Kreft J-U. Mathematical Modeling of Microbial Ecology: Spatial Dynamics of Interactions in Biofilms and Guts. In: Jaykus L-A, Wang HH, Schlesinger LS, editors. Food-Borne Microbes: Shaping the Host Ecosystem. Washington, DC: ASM Press; 2009. pp. 347–377. Available: https://onlinelibrary.wiley.com/doi/abs/10.1128/9781555815479.ch19

2. Hubaux N, Wells G, Morgenroth E. Impact of coexistence of flocs and biofilm on performance of combined nitritation-anammox granular sludge reactors. Water Res. 2015;68: 127–139. doi:10.1016/j.watres.2014.09.036

3. Wanner O, Eberl HJ, Morgenroth E, Noguera DR, Picioreanu C, Rittmann BE, et al. Mathematical modeling of biofilms. London: IWA Publishing; 2006.

4. Lardon LA, Merkey BV, Martins S, Dötsch A, Picioreanu C, Kreft J-U, et al. iDynoMiCS: next-generation individual-based modelling of biofilms. Environ Microbiol. 2011;13: 2416–2434. doi:10.1111/j.1462-2920.2011.02414.x

5. Li B, Taniguchi D, Gedara JP, Gogulancea V, Gonzalez-Cabaleiro R, Chen J, et al. NUFEB: A massively parallel simulator for individual-based modelling of microbial communities. Darling AE, editor. PLOS Comput Biol. 2019;15: e1007125. doi:10.1371/journal.pcbi.1007125

6. Reichert P. Aquasim: A tool for simulation and data analysis of aquatic systems. Water Sci Technol. 1994;30: 21–30.

7. Wanner O, Reichert P. Mathematical modeling of mixed-culture biofilm. Biotechnol Bioeng. 1996;49: 172–184.

8. Reichert P, Wanner O. Movement of solids in biofilms: significance of liquid phase transport. Water Sci Technol. 1997;36: 321–328. Available: https://www.proquest.com/docview/1943242226/abstract/66688444DFA64F6DPQ/1

9. Morgenroth E, Wilderer PA. Influence of detachment mechanisms on competition in biofilms. Water Res. 2000;34: 417–426. doi:10.1016/S0043-1354(99)00157-8

10. Noguera DR, Pizarro GE, Regan JM. Modeling Biofilms. Microbial Biofilms. John Wiley & Sons, Ltd; 2004. pp. 222–249. doi:10.1128/9781555817718.ch13

11. Picioreanu C, Kreft J-U, van Loosdrecht MCM. Particle-based multidimensional multispecies biofilm model. Appl Environ Microbiol. 2004;70: 3024–3040. doi:10.1128/AEM.70.5.3024-3040.2004

12. Rittmann BE, Schwarz AO, Eberl H, Morgenroth E, Peréz J, Van Loosdrecht MCM, et al. Results from the multi-species Benchmark Problem (BM3) using one-dimensional models. Water Sci Technol. 2004;49: 163–168. Available: http://www.iwaponline.com/wst/04911/wst049110163.htm

13. Noguera DR, Picioreanu C. Results from the multi-species Benchmark Problem 3 (BM3) using two-dimensional models. Water Sci Technol. 2004;49: 169–176. Available: http://www.iwaponline.com/wst/04911/wst049110169.htm

14. Kreft J-U. Biofilms promote altruism. Microbiology. 2004;150: 2751–2760. doi:10.1099/mic.0.26829-0

15. Clarke M, Maddera L, Harris RL, Silverman PM. F-pili dynamics by live-cell imaging. Proc Natl Acad Sci. 2008;105: 17978–17981. doi:10.1073/pnas.0806786105
